# Inflammatory Cytokines and SIRT1 Levels in Subcutaneous Abdominal Fat: Relationship With Cardiac Performance in Overweight Pre-diabetics Patients

**DOI:** 10.3389/fphys.2018.01030

**Published:** 2018-08-21

**Authors:** Celestino Sardu, Gorizio Pieretti, Nunzia D'Onofrio, Feliciano Ciccarelli, Pasquale Paolisso, Maria B. Passavanti, Raffaele Marfella, Michele Cioffi, Pasquale Mone, Anna M. Dalise, Franca Ferraraccio, Iacopo Panarese, Antonio Gambardella, Nicola Passariello, Maria R. Rizzo, Maria L. Balestrieri, Gianfranco Nicoletti, Michelangela Barbieri

**Affiliations:** ^1^Department of Medical, Surgical, Neurological, Metabolic and Aging Sciences, University of Campania “Luigi Vanvitelli”, Naples, Italy; ^2^Department of Plastic Surgery, University of Campania “Luigi Vanvitelli”, Naples, Italy; ^3^Department of Precision Medicine, University of Campania “Luigi Vanvitelli”, Naples, Italy; ^4^Department of Clinical, Public and Preventive Medicine, University of Campania “Luigi Vanvitelli”, Naples, Italy

**Keywords:** pre-diabetes, obesity, visceral fat, sirtuin 1, cardiac performance

## Abstract

**Objectives:** In obese patients the superficial adipose tissue expresses cytokines, and sirtuins, that may affect myocardial function. In this study, we investigated the effect of metformin therapy added to a hypocaloric diet on the inflammatory pattern and cardiac performance (MPI) in obese patients with pre-diabetic condition.

**Materials and Methods:** Fifty-eight obese patients that were enrolled for abdominoplastic surgery were divided into patients with pre-diabetic condition (n 40) and normo-glycemic patients (n18). Patients with pre-diabetic condition were randomly assigned to metformin therapy added to a hypocaloric diet (group 1, n 20) or to a hypocaloric diet therapy alone (group 2, n20). Patients with normo-glycemic condition were assigned to a hypocaloric diet therapy.

**Results:** During enrollment, obese patients with a pre-diabetic condition (group 1 and 2) presented higher glucose values, lower values of insulin, and higher values of the homeostasis model for the assessment of insulin resistance (HOMA-IR) than obese patients with normo-glycemic condition(group 3). In addition, they had higher values of C Reactive protein (CRP), interleukin 6 (IL6), and lower values of sirtuin 1(SIRT1). In the 12th month of the follow-up, metformin therapy induced in patients with pre-diabetic condition (group 1) a significant reduction of glucose values, HOMA-IR, and inflammatory markers such as CRP (1.04 ± 0.48 vs. 0.49 ± 0.02 mmol/L, *p* < 0.05), IL6 (4.22 ± 0.45 vs. 3.33 ± 0.34 pg/ml, *p* < 0.05), TNFα (6.95 ± 0.59 vs. 5.15 ± 0.44 pg/ml, *p* < 0.05), and Nitrotyrosine (5,214 ± 0,702 vs. 2,151 ± 0,351 nmol/l, *p* < 0.05). This was associated with a significant reduction of Intima-media thickness (1.01 ± 0.15 vs. 0.86 ± 0.15 mm, *p* < 0.05), Septum (14 ± 2.5 vs. 10.5 ± 2 mm, *p* < 0.05), Posterior wall (11 ± 1.5 vs. 8 ± 1 mm, *p* < 0.05), LV mass (192.5 ± 49.5 vs. 133.2 ± 37.6 g, *p* < 0.05) and of MPI (0.58 ± 0.03 vs. 0.38 ± 0.02, *p* < 0.05). At 12 months of follow-up, group 2 experienced only a reduction of cholesterol (4.15 ± 0.94 vs. 4.51 ± 0.88 mmol/L, *p* < 0.05) and triglycerides (1.71 ± 1.18 vs. 1.83 ± 0.54 mmol/L, *p* < 0.05). At 12 months of follow-up, group 3 experienced a significant reduction of inflammatory markers, and also of echographic parameters, associated with amelioration of myocardial performance. To date, IL6 expression was related to higher values of left ventricle mass (*R*-value 0.272, *p*-value 0.039), and to higher IMT (*R*-value 0.272, *p*-value 0.039), such as those observed for CRP (*R*-value 0.308, *p*-value 0.021), for glucose blood values (*R*-value 0.449, *p*-value 0.001), and for HOMA-IR (*R*-value 0.366, *p*-value 0.005). An inverse correlation was found between subcutaneous fat expression of SIRT1 and myocardial performance index (*R*-value−0.236, *p*-value 0.002).

**Conclusion:** In obese patients with pre-diabetic condition a metformin therapy may reduce inflammation and oxidative stress, and this may be associated with the amelioration of the cardiac performance.

Clinical research trial number: NCT03439592.

## Introduction

In obese patients, visceral fat and superficial adipose tissue are endocrine active tissues that expresses different cytokines, which can cross talk with the cardiovascular system (Marfella et al., 2009). These cytokines, such as tumor necrosis factor-α (TNFα) and interleukin 6 (IL6), together with anti-apoptotic proteins such as sirtuins, play a central role in the regulation of adipose tissue function (Marfella et al., 2009; Houtkooper et al., 2012; Rappou et al., 2016). In fact, the cytokines over expression induces a local dysfunction of the adipose tissue, that is characterized by increased inflammation and oxidative stress, whichis associated to down regulation of mitochondrial biogenesis (Marfella et al., 2009; Houtkooper et al., 2012; Rappou et al., 2016). In this setting, the sirtuins are NAD+ dependent deacetylase, involved in the control of mitochondrial function, energy metabolism, adipocyte hypertrophy (Houtkooper et al., 2012), cardiac regeneration, and cardiac remodeling (Rappou et al., 2016). To date, in obese patients with diabetesthe altered glucose homeostasis induces the up-regulation of inflammatory cytokines, which is consequently associated with sirtuin 1 (SIRT1) down-regulation (Houtkooper et al., 2012). However, this may affect the cardiovascular functions in patients with diabetics vs. normo-glycemic patients, leading to an altered myocardial performance, and to the development of heart failure (Huang et al., 2016; Rappou et al., 2016). In this setting, pre-diabetes is a pathological condition and an intermediate status between normo-glycemic patients and patients with diabetes (Standards of Care, 2017). Pre-diabetics have altered blood glucose and glycated hemoglobin values, which are not in the stipulated range for the diagnosis of diabetes (Standards of Care, 2017). Intriguingly, pre-diabetics have a higher risk of developing myocardial dysfunction, cardiovascular disease, and heart failure, with increasing risk of all causes of mortality (Standards of Care, 2017). Therefore, the altered glucose homeostasis looks to be the key factor conditioning these molecular alterations, and clinical outcomes in patients with pre-diabetes vs. patients with normo-glycemic condition. Conversely, in obese patients the altered inflammation, oxidative stress, and cardiac cellular growth due to the increase of adipose tissue, may affect the myocardial function (Huang et al., 2016; Rappou et al., 2016). Consequently, this pathological condition may be induced and accentuated owing to altered glucose homeostasis in obese patients with pre-diabetic condition vs. patients with normo-glycemic condition. Moreover, in this study we aimed to determine the expression of inflammatory cytokines, oxidative stress factors, SIRT1, and of the cardiovascular parameters in obese patients with pre-diabetes vs. obese patients with normo-glycemic condition. Secondly, we aimed to study the effects of metformin therapy added to a hypocaloric diet vs. placebo in order to control the glucose homeostasis, to revert the inflammatory and oxidative stress status, and the linked worse prognosis. In particular, our attention was focused on obese patients with pre-diabetic condition since, to date, no evidence about the relationship between subcutaneous fat inflammatory pattern/SIRT1 levels and cardiac dysfunction has been described. However, we evaluated the expression of SIRT1 in abdominal fat tissue biopsy at the baseline in the entire study population, and the values of cytokines and nitrotyrosine in peripheral blood of obese patients with pre-diabetic condition as compared to obese patients with normo-glycemic condition at the baseline and at 12 months of follow-up. More in detail, we evaluated the levels of cytokines, nitrotyrosine, and fat tissue SIRT1 expression in patients with pre-diabetic condition treated by a hypocaloric diet-therapy plus metformin (group 1), obese patients with pre-diabetic condition treated by a hypocaloric diet-therapy plus placebo (group 2), and obese patients with normo-glycemic condition treated by a hypocaloric diet-therapy (group 3). Thereafter, the levels of cytokines and fat tissue SIRT1 expression were correlated to clinical variables and their changes at follow-up, such as intima-media wall thickness (IMT), left ventricle (LV) mass, left ventricle ejection fraction (LVEF), and myocardial performance index (MPI) as an index of myocardial performance (Marfella et al., 2009).

## Materials and methods

### Research design and methods

We prospectively enrolled 58 obese patients with standard symptoms requiring abdominoplastic surgery in a placebo-controlled study, conducted from January 2015 to January 2018 at the University of Campania “Luigi Vanvitelli” (6). Obesity was diagnosed as body mass index (BMI) > 30 (WHO, 2000). All 58 patients underwent abdominoplastic surgery, and after treatment received a hypocaloric diet. The mean recommended daily caloric intake was 1300 kcal, ranging from 1,250 to 1,350 kcal. The recommended composition of the dietary regimen was 55% carbohydrates, 30% lipid, and 15% protein. Forty obese patients had pre-diabetes according to international guidelines diagnostic criteria (Standards of Care, 2017). Patients with prediabetes were diagnosed by evidence of fasting plasma glucose values of ≥5.6 mmol/L but < 7.0 mmol/L (100–125 mg/dL; impaired fasting glucose [IFG]), a 2-h glucose value of ≥7.8 mmol/L but < 11.1 mmol/L during a 75 g oral glucose tolerance test (GTT) (140–199 mg/dL; impaired glucose tolerance [IGT]), or a plasma hemoglobin (Hb) A1c value of ≥5.7% but < 6.5% (Standards of Care, 2017). Patients with Pre-diabetic condition were randomly divided in two groups: group 1 (*n* = 20) treated by a hypocaloric diet added to metformin therapy vs. group 2 (*n* = 20) treated by a hypocaloric diet plus placebo. The patients with pre-diabetic condition in group 1 received metformin 850 mg twice a day, while the patients with pre-diabetic condition in group 2 received a placebo. Eighteen patients were obese normo-glycemics (WHO, 2000; Sozer et al., 2007; Lang et al., 2015; Standards of Care, 2017). All study groups volunteered for repeated clinical evaluations and laboratory analyses as well as echocardiography. In the follow-up period, patients were treated with a multidisciplinary approach consisting of diet, exercise, and behavioral and nutritional counseling as previously described (Marfella et al., 2009). The enrolled patients were followed up quarterly on an outpatient basis until 12 months. Exclusion study criteria were: diagnosis of type 2 diabetes, cardiovascular disease, psychiatric problems, a history of alcohol abuse, smoking, or any hypoglycemic medication assumption. All patients had normal results used as laboratory data (urea nitrogen, creatinine, electrolytes, liver function tests, uric acid, thyroxin, and complete blood count), chest x-rays, and electrocardiograms. All patients were evaluated at the baseline, and at 12 months follow-up. Each patient provided informed written consent to participate in this study, which was approved by the institutional committee of ethical practice of our institution. The patients subscribed a separate informed consent to undergo abdominoplasty.

### Anthropometric parameters

We measured the height and the weight of all patients, and BMI was calculated as weight in kilograms divided by the square of height in meters (Marfella et al., 2009). Waist hip ratio (WHR) was calculated as waist circumference in centimeters divided by hip circumference in centimeters, and as an index of central obesity (Marfella et al., 2009). As previously reported (Marfella et al., 2009), in all obese patients we evaluated insulin blood levels, and the homeostasis model for the assessment of insulin resistance (HOMA-IR).

### Echocardiography

All patients underwent a transthoracic full 2-dimensional and Doppler echocardiography assessment according to the American Society of Echocardiography recommendations (Lang et al., 2015). The exam was performed at the baseline and after 12 months. All scans and measurements were performed by a single experienced physician trained in cardiovascular ultrasound, using a Philips iE33 echocardiograph (Eindhoven, The Netherlands). Two independent investigators performed the analysis of echocardiography tracings blinded to treatment groups. Left ventricular mass was calculated and normalized by both body surface area (BSA)and by height squared corrected for the effect of overweight (de Simone et al., 1992). As described previously, we calculated the left ventricle ejection fraction, as an index of cardiac pumping (8). Left ventricle ejection fraction was calculated, dividing the stroke volume by the volume of blood collected in the left ventricle at the end of diastolic filling as end-diastolic volume (Lang et al., 2015). The stroke volume was the fraction of chamber volume ejected in systole as the difference between end diastolic volume and end systolic volume (Lang et al., 2015). Subsequently, we measured Doppler velocities and time intervals from mitral inflow and left ventricular outflow recordings. We measured mitral early diastolic flow deceleration time, as the time interval between the peak of early diastolic velocity and the end of early diastolic flow, and total systolic time interval from the cessation of one mitral flow to the beginning of the following mitral inflow (Marfella et al., 2009; Lang et al., 2015). The ratio of velocity time intervals of mitral early and late diastolic flows was then calculated (Lang et al., 2015). Other myocardial Doppler indexes and intervals were left ventricle isovolumetric relaxation time (IRT), left ventricle ejection time (ET), and left ventricle isovolumetric contraction time (ICT) (Marfella et al., 2009; Lang et al., 2015). In detail, IRT was the time interval from cessation of left ventricular outflow to onset of mitral inflow (Lang et al., 2015). ET was the left ventricle ejection time, as an interval from the onset and cessation of left ventricular outflow (Lang et al., 2015). ICT was an interval calculated by subtracting ET and IRT from the total systolic time interval (Lang et al., 2015). Therefore, from these indexes we obtained the myocardial performance index. In fact, the myocardial performance index was calculated by using the formula MPI = (IRT + ICT)/ET (Marfella et al., 2009; Lang et al., 2015). To date, MPI is a parameter that includes both systolic and diastolic time intervals, and it is a single parameter that can be used for the assessment of cardiac dysfunction (Marfella et al., 2009; Lang et al., 2015).

### Measuring the IMT of the carotid artery

B-mode ultrasound imaging of the carotid arteries was performed in each patient at the baseline and at 12 months after a Philips iE33 echocardiograph (Eindhoven, The Netherlands) with a 7-MHzlinear array transducer was used to clearly display both the blood–intima and media–adventitia boundaries on the far walls of the arteries. The lumen of the arteries was maximized using gain settings to optimize image quality. The protocol for measuring carotid artery IMT consisted of scanning the right and left common carotid arteries longitudinally in the 5- to 20-mm segment proximal to the carotid bulb and in areas free of plaques (Marfella et al., 2009). IMT measurements were performed offline on a personal computer, to locate the lumen-intima and media–adventitia echographic boundaries. The presence of plaques was defined as localized echo structures encroaching into the lumen of the vessel for which the distance between the media-adventitia interface and the internal side of the lesion was >1.5 mm. A single experienced physician trained in vascular ultrasound performed all scans and IMT measurements, and the intra observer coefficient of variation for C-IMT was < 3%.

### Analyses of blood samples

Serum samples for cytokine levels were stored at temperatures under 80°C until assayed. Serum concentrations of TNFα, IL6, and Nitrotyrosine were determined in duplicate using a highly sensitive quantitative sandwich enzyme assay (ELISA, Quantikine HS; R&D Systems, Minneapolis, MN). Venous blood samples were drawn for nitrotyrosine evaluation. Nitrotyrosine plasma concentration was assayed by ELISA, after an overnight fast, at breakfast time, and before the intervention. Assays for serum total and high-density lipoprotein cholesterol, triglyceride, and glucose levels were performed in the hospital's chemistry laboratory. Plasma insulin levels were assayed by radioimmunoassay (Ares, Serono, Italy). Insulin resistance in the fasting state was assessed with homeostasis model assessment (HOMA) and calculated with the following formula: fasting plasma glucose (millimoles per liter) times fasting serum insulin (microunits per milliliter) divided by 25, as described previously (Marfella et al., 2009).

### Abdominal dermolipectomy

Patients underwent conventional abdominoplasty surgical procedure, with umbiliculum transposition and cutaneous adipose mass tissue excision ranging from 200 g, as previously reported (Marfella et al., 2009). Patients were mobilized 24 h after surgery; anti-inflammatory therapy (non-steroidal anti-inflammatory drugs) was suspended after 48 h and patients were discharged 72 h following antibiotic therapy.

## Analyses of adipose tissue

After surgery, the specimens were cut parallel to the long axis into four different parts for the different works-ups. The first part was frozen in liquid nitrogen for the following enzyme-linked immunosorbent assay analysis. A portion of the other specimens was immediately immersion fixed in 10% buffered formalin. Sections were serially cut at 5 μm, mounted on lysine-coated slides, and stained with hematoxylin/eosin. Specimens were analyzed by light microscopy.

### Western blot analysis

For detection of SIRT1 expression in adipose abdominal superficial tissue, samples (200 mg) from obese patients with normo-glycemic condition and obese patients with pre-diabetic condition were cut into small pieces and homogenization was performed by adding 600 μL of 2D lysis buffer (7 mM urea, 2 mMthiourea, 4% CHAPS [3-([3-cholamidopropyl] dimethylammonio)-1-propane sulfonate] buffer and 30 mMTris-HCl, pH 8.8 to tissues. Tissues homogenized with a Precellys 24 system (Bertin Technologies, Montignyle-Bretonneux, France) were centrifuged at 800 × g for 10 min at 4°C to collect the supernatant. Proteins were then precipitated by adding 100% cold methanol. Protein extracts (80 μg) were separated by SDS-polyacrylamide gel electrophoresis (7%) and transferred onto nitrocellulose membranes by a Trans-Blot Turbo Transfer System (BioRad). Membranes were blocked in 5% w/v milk, 1 X Tris-buffered saline (TBS), 0.1% Tween-20 at 25°C for 2 h with gentle shaking, and then incubated over night at 4°C with antibodies against SIRT1 (1:1,000) (rabbit monoclonal, ab32441, Abcam, Cambridge, UK) or NF-κB (1:1,000) (rabbit monoclonal, C22B4, Cell Signaling Technology, Danvers, MA) or Fas (rabbit monoclonal, ab82419, Abcam, Cambridge, UK). After incubation with secondary antibody (1:10,000), membranes were washed three times and bands were detected by the enhanced chemiluminescence kit (Immobilon Western, Chemiluminescent HRP Substrate, Millipore, Billerica, MA 01821, USA) and analyzed by using a Scan LKB (Amersham Pharmacia). Normalization of results was ensured by incubating the nitrocellulose membranes in parallel with the alpha-tubulin antibody (1:1,000) (mouse monoclonal, 3,873, Cell Signaling Technology, Danvers, MA).

### Enzyme-linked immunosorbent assay (ELISA)

For detection of IL6 and TNF-a levels in adipose abdominal superficial tissue samples from patients with obese normo-glycemic and obese pre-diabetic conditions, we used the enzyme-linked immunosorbent assay (ELISA) kit. ELISA is a commonly used enzyme-based assay for the detection of a variety of analytes (Quantikine HS; R&D Systems, Minneapolis, MN).

### Immunohistochemistry

After the surgical procedure, other samples that were not used for ELISA were immediately frozen in isopentane and cooled in liquid nitrogen. Serial sections were incubated with specific antibodies anti–TNFα and anti-IL6 (R&D, Minneapolis, USA), anti-SIRT1 (Abcam, Cambridge, UK), anti-Fas (Abcam, Cambridge, UK), anti-nitrotyrosine (Santa Cruz, California, USA), and anti-NFKb (Cell Signaling Technology, Danvers, MA). However, the positive-cells were counted individually and expressed as the number of cells per square millimeter of section area and determined by computer-aided planimetry (defined in later text). Furthermore, we determined the area occupied by anti–TNFα and anti-IL6 (R&D, Minneapolis, USA), anti-SIRT1 (Abcam, Cambridge, UK), anti-Fas (Abcam, Cambridge, UK), anti-nitrotyrosine (Santa Cruz, California, USA), and anti-NFKb (Cell Signaling Technology, Danvers, MA) -positive–rich areas. Analysis of experiments was performed with a personal computer based 24-bit color image-analysis system. In brief, electronic images were digitized with a Leicacolor video camera (Leica Microsystem, Milano, Italy). A color threshold mask for immunostaining was defined to detect the red color by sampling, and the same threshold was applied to all specimens. The percentage of the total area with positive color for each section was recorded. Analysis of immunohistochemistry was performed with a personal computer-based quantitative 24-bit color image analysis system (IM500, Leica Microsystem).

## Statistical analysis

Data were presented as group mean ± SD. A One-way analysis of variance (ANOVA) was used to compare baseline data, followed by Scheffe's test for pair wise comparisons. Simple and partial correlations were used to evaluate relationships between variables. A linear regression analysis was performed and corrections made with study variables (CRP, IL6, TNFα, SIRT1, cholesterol, creatinine, insulin, HOMA-IR, systolic arterial pressure, glucose) and the clinical study outcomes such as left ventricle mass, left ventricle ejection fraction, myocardial performance index and carotid artery intima-media wall thickness. In detail, these study variables were correlated to the delta values of study outcomes, that represented changes between follow-up vs. baseline values. The presented data were a combination of group 1, group 2 and group 3, and we performed a partial correlation with groups and fasting glucose as a confounder. However, for each study outcome we calculated the *R*-value and the *p*-value. A value of *P* < 0.05 was considered significant. Statistical analysis was performed using the SPSS software package for Windows 17.0 (SPSS Inc., Chicago Illinois). We calculated a sample size with 15 participants for each group, with estimated 80% power to detect a change of 0.015 between the mean MPI of the placebo-treated and actively treated groups, at a 5% level of significance. A 20% loss due to early withdrawals and/or non-evaluable measurements was assumed, which combined with the effect of stratification on analysis, resulted in the requirement to recruit at least 18 patients per treatment group.

## Results

### Clinical characteristics of study population at enrollment

**Figure 1:**
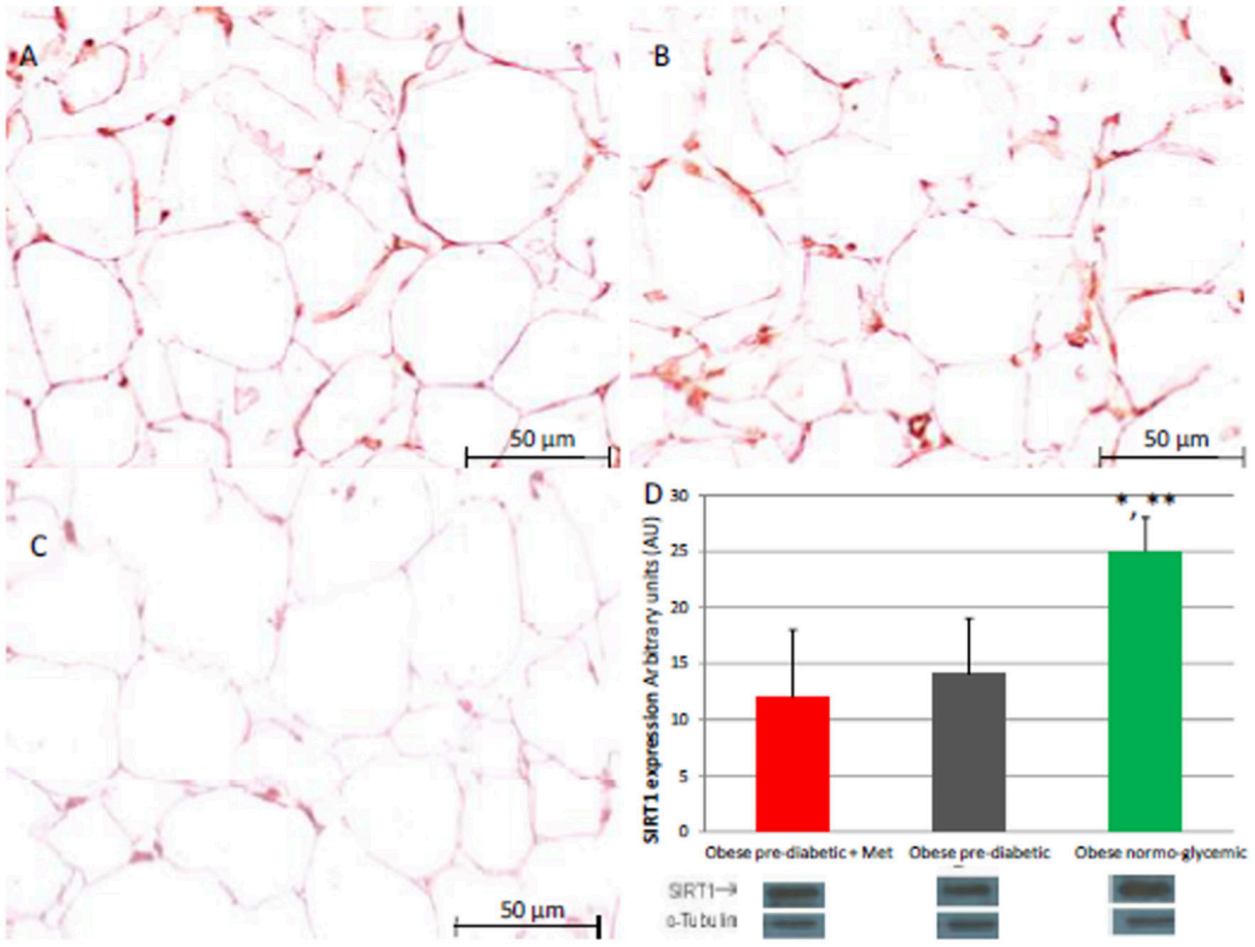
Immunohistochemical analysis for Sirtuin 1 (SIRT1). Representative images of SIRT1 detection in subcutaneous abdominal fat from obese patients with pre-diabetic condition and obese patients with normo-glycemic condition. Immunoistochemistry of obese patients with pre-diabetic condition in metformin therapy (group 1, left upper part, **A**) vs. obese patients with pre-diabetic condition in placebo therapy (group 2, right upper part, **B**) vs. obese patients with normo glycemic condition (group 3, in the left inferior part, **C**); In the right inferior part **(D)**, bar graph expression and matched western blot expression of SIRT1 in obese patients with pre-diabetic condition in metformin therapy (group 1, red color), obese patient with pre-diabetic condition in placebo therapy (group 2, gray color), and obese patients with normo glycemic condition(group 3, green color). Protein levels determined by Western blot analysis of adipose abdominal tissue homogenates from obese patients with normo-glycemic condition and obese patients with pre-diabetic condition. Inset, representative image of Western blot analysis **(D)**. Lanes 1, obese patient with pre-diabetic condition in metformin therapy (group 1). Lanes 2, obese patient with pre-diabetic condition in placebo therapy (group 2). Lanes 3, obese patient with normo-glycemic condition (group 3). Results are mean ± SD of 5 independent experiments. Group 1 vs. group 3, ^*^P <0.05; group 2 vs. group 3, ^**^p <0.05.

**Figure 2:**
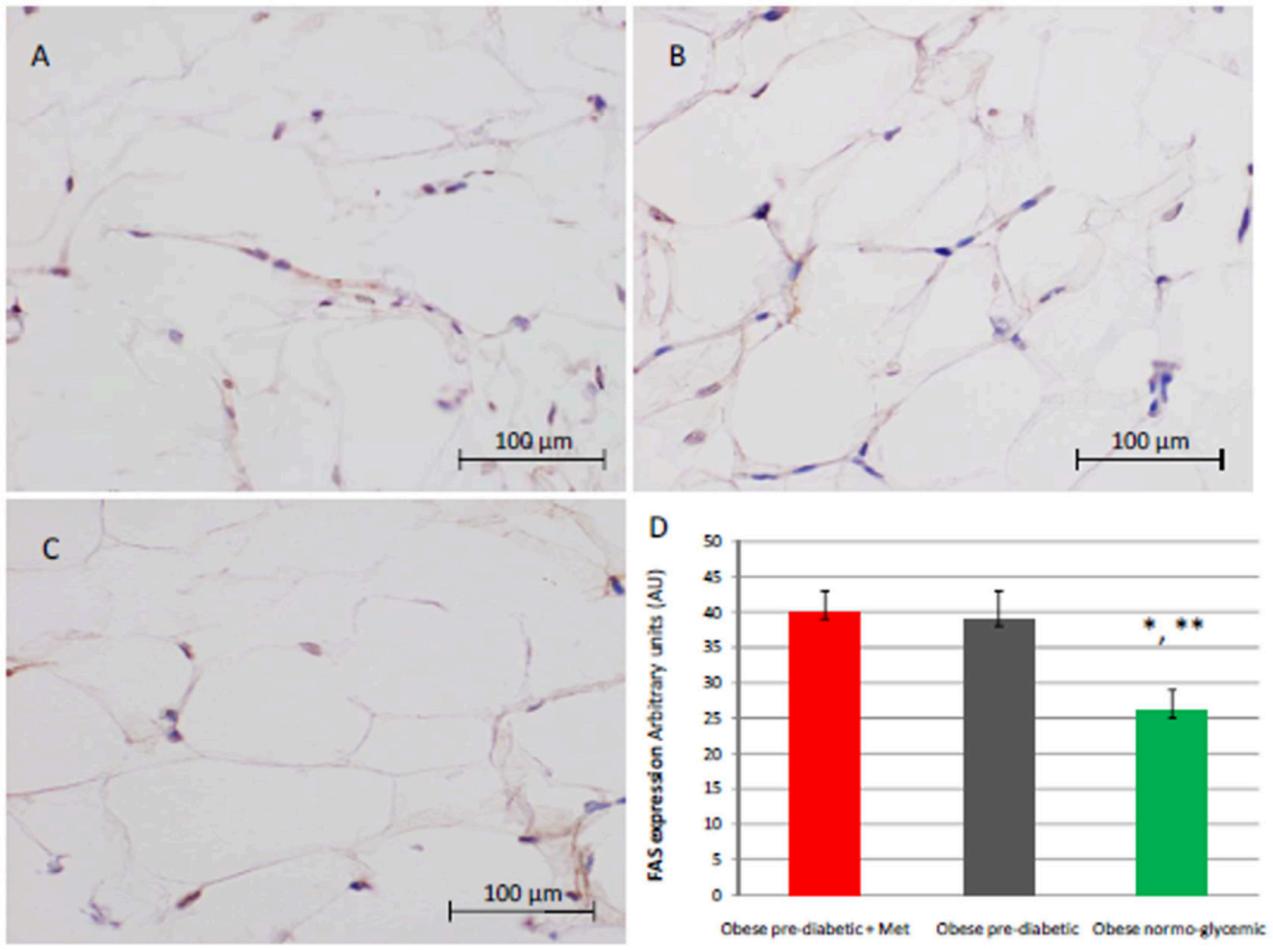
Immunohistochemical analysis for Fas. Representative images of Fas detection in subcutaneous abdominal fat from obese patient with pre-diabetic condition and obese patient with normo-glycemic condition. Immunoistochemistry of obese patient with pre-diabetic condition in metformin therapy (group 1, left upper part, **A**) vs. obese patient with pre-diabetic condition in placebo therapy (group 2, right upper part, **B**) vs. obese patient with normo glycemic condition (group 3, in the left inferior part, **C**); In the right inferior part **(D)**, expression of FAS in obese patient with pre-diabetic condition in metformin therapy (group 1, red color), obese patient with pre-diabetic condition in placebo therapy (group 2, gray color), and in obese patient with normo glycemic condition (group 3, green color). Results are mean ± SD of 5 independent experiments. Results are mean ± SD of 5 independent experiments. Group 1 vs. group 3, ^*^P <0.05; group 2 vs. group 3, ^**^p <0.05.

**Figure 3:**
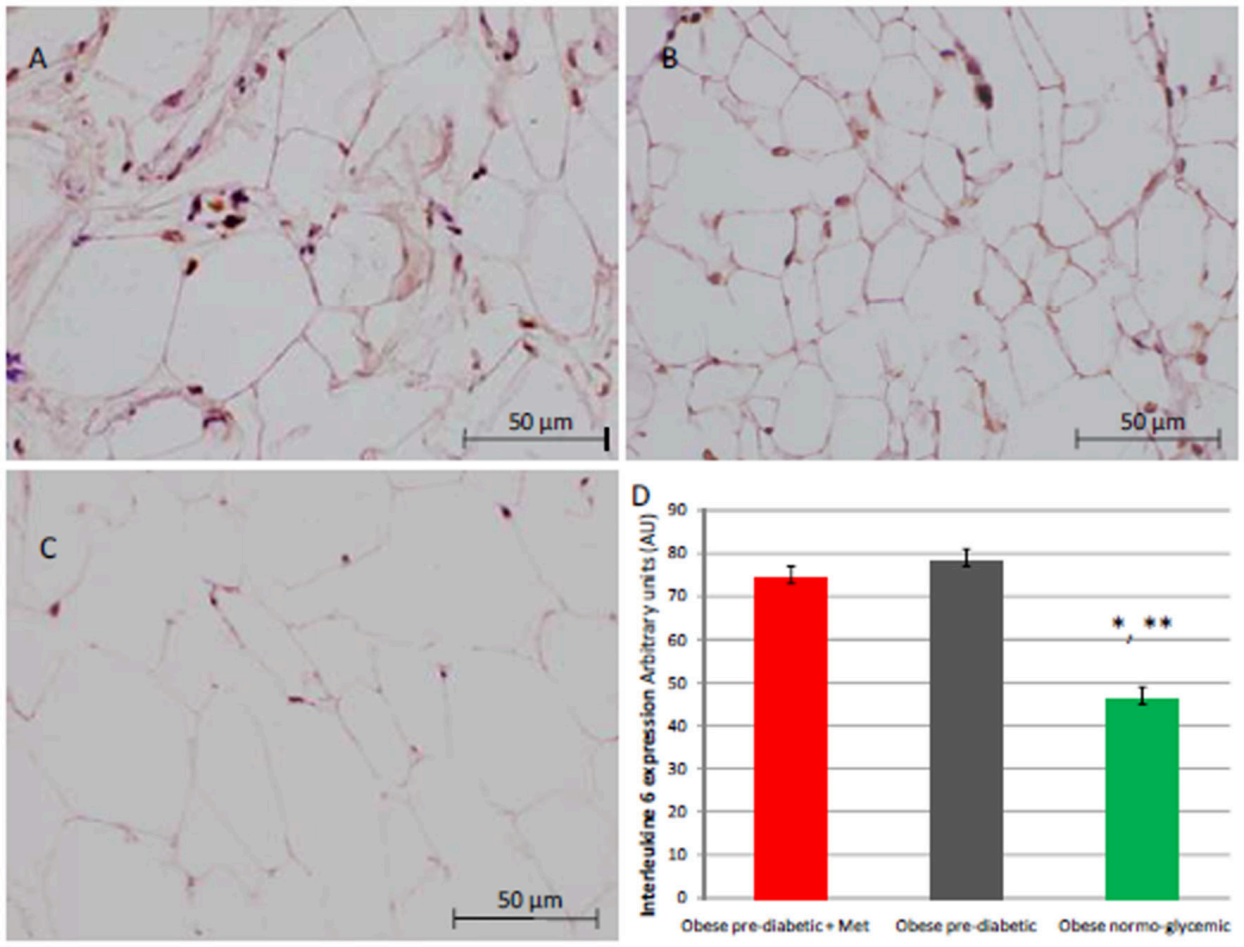
Immunohistochemical analysis for Interleukine 6 (IL6). Representative images of IL6 immunohistochemical detection in subcutaneous abdominal fat from obese patients with pre-diabetic condition and obese patient with normo-glycemic condition. Immunoistochemistry of obese patient with pre-diabetic condition in metformin therapy (group 1, left upper part, **A**) vs. obese patient with pre-diabetic condition in placebo therapy (group 2, right upper part, **B**) vs. obese patient with normo glycemic condition (group 3, in the left inferior part, **C**); In the right inferior part **(D)**, expression of IL6 in obese patient with pre-diabetic condition in metformin therapy (group 1, red color), obese patient with pre-diabetic condition in placebo therapy (group 2, gray color), and in obese patient with normo glycemic condition (group 3, green color). Results are mean ± SD of 5 independent experiments. Group 1 vs. group 3, ^*^P <0.05; group 2 vs. group 3, ^**^p <0.05.

**Figure 4:**
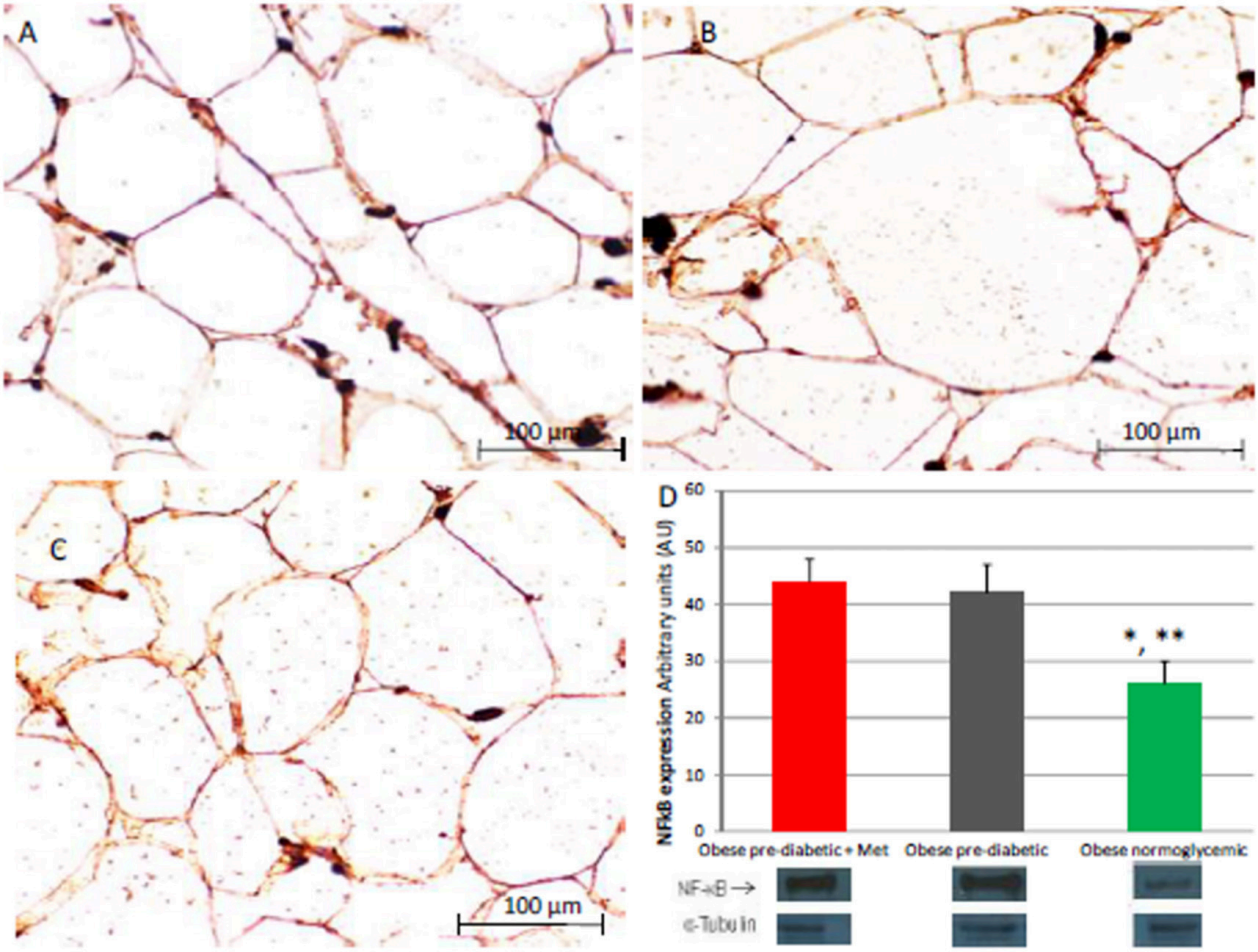
Immunohistochemical detection of Nuclear Factor kappa-light-chain-enhancer of activated B cells (NFkB). Immunohistochemistry of obese patients with normo glycemic condition (group 3, in left upper part, **A**) vs. obese patient with pre-diabetic condition in metformin therapy (group 1, right upper part, **B**) vs. obese patient with pre-diabetic condition in placebo therapy (group 2, left lower part, **C**); In the right inferior part **(D)**, bar graph expression and matched western blot expression of NFkB in obese patient with pre-diabetic condition in metformin therapy (group 1, red color), obese patient with pre-diabetic condition in placebo therapy (group 2, gray color), and obese patient with normo glycemic condition (group 3, green color). Protein levels are determined by Western blot analysis of adipose abdominal tissue homogenates from obese patients with normo-glycemic conditionand obesepatients with pre-diabetic condition. Inset, representative image of Western blot analysis **(D)**. Lanes 1, obese patient with pre-diabetic condition in metformin therapy (group 1). Lanes 2, obese patient with pre-diabetic condition in placebo therapy (group 2). Lanes 3, obese patient with normo-glycemic condition (group 3). Results are mean ± SD of 5 independent experiments. Group 1 vs. group 3, ^*^P <0.05; group 2 vs. group 3, ^**^p <0.05.

**Figure 5:**
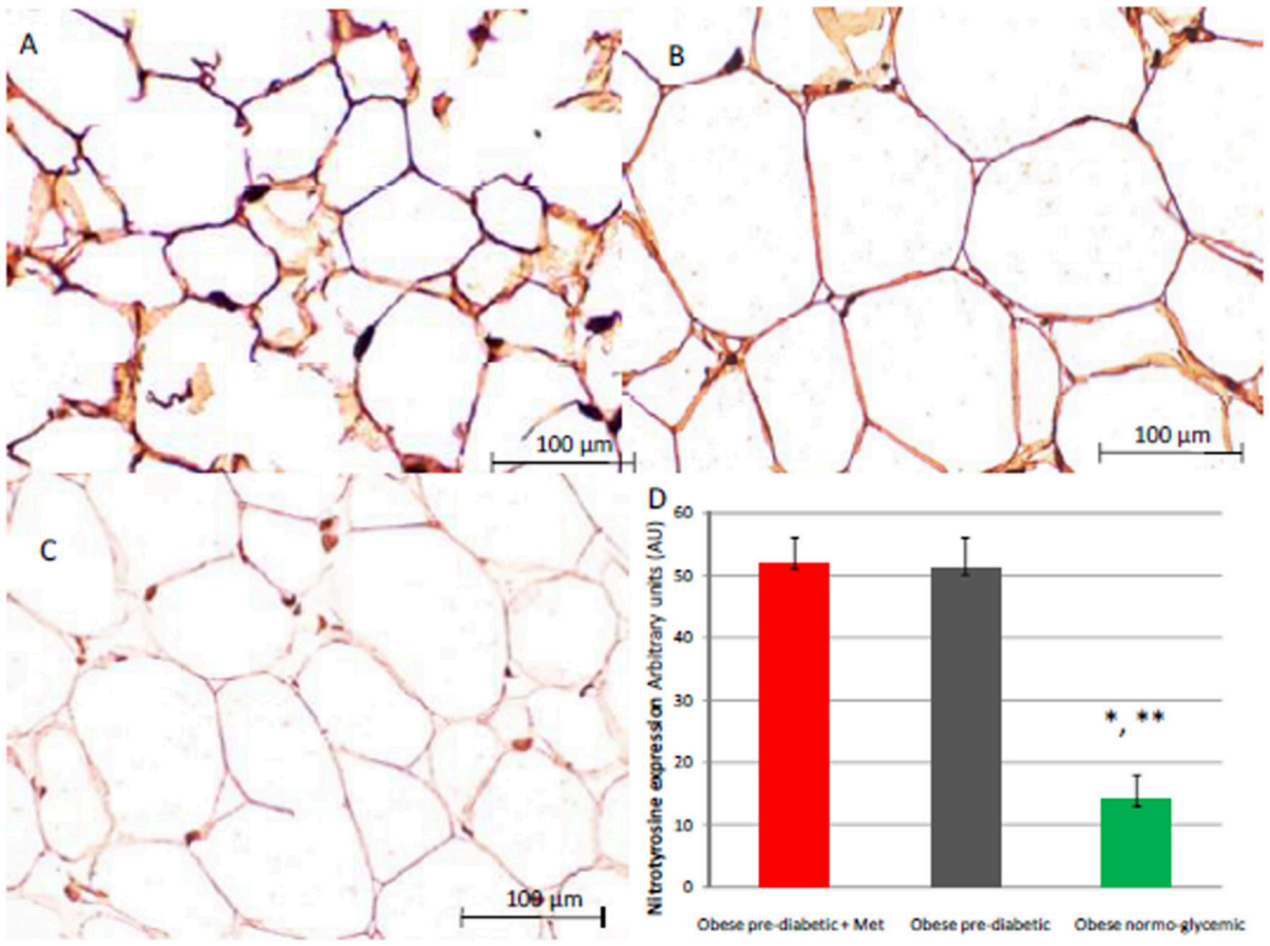
Immunohistochemical detection of Nitrotyrosine. Representative images of Nitrotyrosine immunohistochemical detection in subcutaneous abdominal fat from obese patients with pre-diabetic condition and obese patients with normo-glycemic condition. Immunoistochemistry of obese patients with normo glycemic condition (group 3, in theleft upper part, **A**) vs. obese patients with pre-diabetic condition in metformin therapy (group 1, right upper part, **B**) vs. obese patient with pre-diabetic condition in placebo therapy (group 2, left lower part, **C**); In the right inferior part **(D)**, expression of Nitrotyrosyne in obese patients with pre-diabetic condition in metformin therapy (group 1, red color), obese patient with pre-diabetic condition in placebo therapy (group 2, gray color), and obese patients with normo glycemic condition (group 3, green color). Results are mean ± SD of 5 independent experiments. Group 1 vs. group 3, ^*^P <0.05; group 2 vs. group 3, ^**^p <0.05.

**Figure 6:**
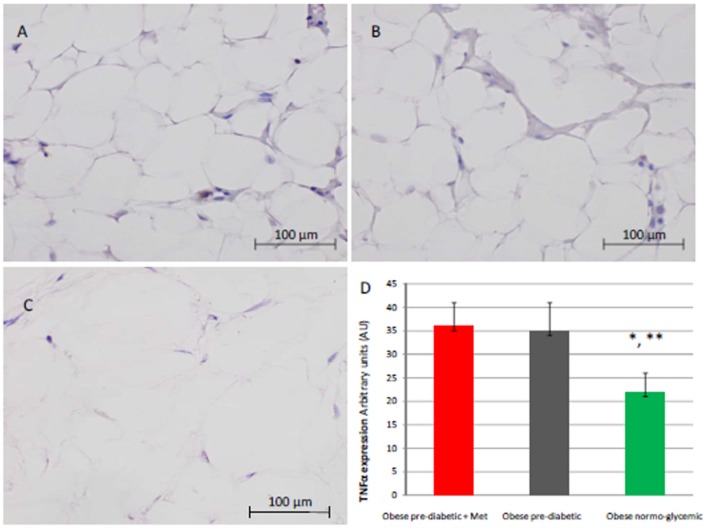
Immunohistochemical detection of Tumor Necrosis factor alpha (TNFα). Representative images of TNFαimmunohistochemical detection in subcutaneous abdominal fat from obese patient with pre-diabetic condition and obese patient with normo-glycemic condition. Immunoistochemistry of obese patient with normo glycemic condition (group 3, in left upper part, **A**) vs. obese patient with pre-diabetic condition in metformin therapy (group 1, right upper part, **B**) vs. obese patient with pre-diabetic condition in placebo therapy (group 2, left lower part, **C**); In the right inferior part **(D)**, expression of TNFα in obese patient with pre-diabetic condition in metformin therapy (group 1, red color), obese patient with pre-diabetic condition in placebo therapy (group 2, gray color), and in obese patient with normo glycemic condition (group 3, green color). Results are mean ± SD of 5 independent experiments. Group 1 vs. group 3, ^*^P <0.05; group 2 vs. group 3, ^**^p <0.05.

At enrollment, obese patients with pre-diabetic condition in groups 1 and 2 vs. normo-glycemic obese patients (group 3) presented higher values of glucose (6.64 ± 0.14 vs. 5.34 ± 0.57 mmol/L, and 6.73 ± 0.24 vs. 5.34 ± 0.57 mmol/L, *p* < 0.05), lower values of insulin (19.7 ± 1.9 vs. 22.6 ± 1.9 μU/mL, and 20.1 ± 1.8 vs. 22.6 ± 1.9 μU/mL, *p* < 0.05), and higher values of HOMA-IR (5.1 ± 0.72 vs. 4.1 ± 0.28, and 4.9 ± 0.68 vs. 4.1 ± 0.28, *p* < 0.05) Table 1. At enrollment, obese patients with prediabetic condition in groups 1 and 2 vs. obese patients with normo-glycemic condition(group 3) presented higher values of creatinine (98.6 ± 4.4 vs. 78.3 ± 2.6 mmol/L, and 101.2 ± 3.5 vs. 78.3 ± 2.6 mmol/L, *p* < 0.05), and higher values of inflammatory markers, such as CRP (1.04 ± 0.48 vs. 0.85 ± 0.38 mmol/L, and 1.03 ± 0.43 vs. 0.85 ± 0.38 mmol/L, *p* < 0.05), IL6 (4.22 ± 0.45 vs. 3.53 ± 0.43 pg/ml, and 4.10 ± 0.39 vs. 3.53 ± 0.43 pg/ml, *p* < 0.05), TNFα (6.95 ± 0.59 vs. 5.51 ± 1.09 pg/ml, and 6.75 ± 0.53 vs. 5.51 ± 1.09 pg/ml, *p* < 0.05), and Nitrotyrosine (5,214 ± 0,702 vs. 1,211 ± 0,205 nmol/L, and 5,092 ± 0,651 vs. 1,211 ± 0,205 nmol/L, *p* < 0.05) Table 1. During enrollment patients with pre-diabetic condition(group 1) vs. patients with normo-glycemic condition presented higher values of Intima-media thickness (1.01 ± 0.15 vs. 0.85 ± 0.14 mm, *p* < 0.05), whereas they did not have differences with other echocardiographic parameters, and cardiac functional indexes, such as LVEF and MPI Table 1. The Figures 1–6 represent at the baseline the local adipose tissue expression in arbitrary units, and the mean ± SD of 5 independent experiments, of SIRT1 (0.76 ± 0.02 vs. 0.91 ± 0.04, *p* < 0.05; 0.68 ± 0.03 vs. 0.91 ± 0.04, *p* < 0.05), NFkB (1.04 ± 0.02 vs. 0.98 ± 0.02, *p* < 0.05; 1.21 ± 0.03 vs. 0.98 ± 0.02, *p* < 0.05), TNFα (0.36 ± 0.05 vs. 0.22 ± 0.04, *p* < 0.05; 0.35 ± 0.06 vs. 0.22 ± 0.04, *p* < 0.05), IL6 (0.74 ± 0.03 vs. 0.46 ± 0.03, *p* < 0.05; 0.78 ± 0.03 vs. 0.46 ± 0.03, *p* < 0.05), Fas (0.40 ± 0.03 vs. 0.26 ± 0.03, *p* < 0.05; 0.39 ± 0.04 vs. 0.26 ± 0.03, *p* < 0.05), and Nitrotyrosine (0.52 ± 0.04 vs. 0.14 ± 0.04, *p* < 0.05; 0.51 ± 0.05 vs. 0.14 ± 0.04, *p* < 0.05), respectively comparing group 1 vs. group 3, and group 2 vs. group 3, Figures 1–6.

**Table 1:** Clinical characteristics of the study population at baseline.

**Study variables**	**Group 1 (n 20)**	**Group 2 (n 20)**	**Group 3 (n 18)**	***P*-value**
**CLINICAL VARIABLES**				
Age	40.5 ± 7	40.5 ± 6	39.0 ± 8	/
Male (%)	5 (25)	6 (30)	5 (27.8)	/
BMI (Kg/m^2^)	33.1 ± 2.7	33.5 ± 2.6	33.7 ± 2.4	/
Systolicarterial pressure (mmHg)	133 ± 11	129 ± 12	126 ± 10.3	/
Diastolicarterial pressure (mmHg)	82 ± 2.3	84 ± 2.1	85 ± 2.1	/
Heart rate (beats for minute)	72 ± 9	72 ± 10	69 ± 8	/
WHR	0.91 ± 0.006	0.91 ± 0.005	0.91 ± 0.001	/
HOMA-IR	5.1 ± 0.72	4.9 ± 0.68	4.1 ± 0.28	<0.05^**^, < 0.05^***^
Insulin (μU/mL)	19.7 ± 1.9	20.1 ± 1.8	22.6 ± 1.9	<0.05^**^, <0.05^***^
Glucose (mmol/L)	6.64 ± 0.14	6.73 ± 0.24	5.34 ± 0.57	<0.05^**^, <0.05^***^
Cholesterol(mmol/L)	4.33 ± 0.86	4.51 ± 0.88	4.66 ± 1.02	/
HDL(mmol/L)	1.73 ± 0.44	1.83 ± 0.39	1.78 ± 0.41	/
LDL(mmol/L)	3.31 ± 0.61	3.33 ± 0.57	3.17 ± 0.59	/
Triglycerides(mmol/L)	1.89 ± 0.44	1.83 ± 0.54	1.61 ± 0.31	/
Creatinine (mmol/L)	98.6 ± 4.4	101.2 ± 3.5	78.3 ± 2.6	<0.05^**^, <0.05^***^
**BIOHUMORAL MARKERS**				
CRP (mmol/L)	1.04 ± 0.48	1.03 ± 0.43	0.85 ± 0.38	<0.05^**^, <0.05^***^
IL6(pg/ml)	4.22 ± 0.45	4.10 ± 0.39	3.53 ± 0.43	<0.05^**^, <.05^***^
TNFα (pg/ml)	6.95 ± 0.59	6.75 ± 0.53	5.51 ± 1.09	<0.05^**^, <0.05^***^
Nitrotyrosine (nmol/l)	5.214 ± 0.702	5.309 ± 0.651	1.211 ± 0.205	<0.05^**^, <0.05^***^
**ADIPOSE TISSUE MARKERS**				
SIRT1 (arbitraryunits)	0.65 ± 0.29	0.63 ± 0.23	0.85 ± 0.10	<0.05^**^, <0.05^***^
NFKb (arbitraryunits)	1.08 ± 0.12	1.10 ± 0.09	0.91 ± 0.06	<0.05^**^, <0.05^***^
FAS (arbitraryunits)	217.51 ± 85.09	224.45 ± 95.15	69.93 ± 23.84	<0.05^**^, <0.05^***^
IL6(arbitraryunits)	74.08 ± 3.11	78.12 ± 3.08	46.18 ± 3.04	<0.05^**^, <0.05^***^
TNFα(arbitrary units)	36.51 ± 5.16	35.72 ± 6.21	22.37 ± 4.10	<0.05^**^, <0.05^***^
Nitrotyrosine (arbitraryunits)	52.82 ± 4.28	51.68 ± 5.12	14.18 ± 4.15	<0.05^**^, <0.05^***^
**ECHOCARDIOGRAPHICPARAMETERS**				
Intima-media tickness	1.01 ± 0.15	1.03 ± 0.18	0.85 ± 0.14	<0.05^**^, <0.05^***^
LVTDd (mm)	56 ± 3.8	55 ± 4.1	54 ± 4.6	/
LVTSd (mm)	34 ± 4.4	32 ± 4.8	31 ± 6.7	/
LVEF (%)	54 ± 6	54 ± 7	53 ± 6	/
LAD (mm)	45 ± 6	43 ± 6	42 ± 2	/
Septum (mm)	14 ± 2.5	14 ± 2.2	13.5 ± 2.6	/
Posteriorwall (mm)	11 ± 1.5	11 ± 1	11 ± 1	/
MPI	0.58 ± 0.03	0.57 ± 0.03	0.57 ± 0.03	/
LV mass (g)	192.5 ± 49.5	191.7 ± 49.7	203.7 ± 48.4	/
LV mass/BSA (g/m^2^)	84.06 ± 21.62	82.62 ± 21.42	90.13 ± 21.42	/
LV mass/h (m^2^)	69 ± 17.75	67.03 ± 17.38	72.23 ± 17.16	/
**DRUG THERAPY**				
ACE inhibitors (%)	10 (50)	9 (45)	9 (50)	/
ARS blockers (%)	5 (25)	5 (25)	5 (28)	/
Calciumchannelsblockers (%)	2 (10)	2 (10)	2 (11.1)	/
Loopdiuretics (%)	2 (10)	2 (10)	2 (11.1)	/
Metformin (%)	20 (100%)	0	0	
Statin (%)	8 (40)	9 (45)	7 (39)	/
Thiazides (%)	5 (25)	6 (30)	5 (27.8)	/

Clinical characteristics of the study population for obese patients with pre-diabetic condition (groups 1 and 2), and obese patients with normo-glycemic condition (group 3) at baseline. In this table the study variables of the study population of 58 patients are reported divided into three groups at baseline: groups 1, 20 obese patients with pre-diabetic condition treated with a hypocaloric diet added to metformin; group 2, 20 obese patients with pre-diabetic condition treated with a hypocaloric diet added to placebo; group 3, 18 obese patients with normo-glycemic condition treated with a hypocaloric diet. In the study the variables (first column to the left) reported are clinical variables, biohumoral markers, echocardiographic parameters, and drug therapy for each group of patients. We reported fasting glucose and lipid values. ACE is angiotensin-converting enzyme; ARS is angiotesin renin system; CRP, C reactive protein; h is height; HOMA-IR, homeostasis model for the assessment of insulin resistance; IL6, interleukine 6; LAD is the left atrium diameter; LV is the left ventricle; LVEF is the left ventricle ejection fraction; LVTDd is the left ventricle telediastolic diameter; LVTDs is the left ventricle telesystolic diameter; MPI is the myocardium performance index; SIRT 1, sirtuin 1; TNFα is tumor necrosis factor alpha; WHR, waist hip ratio. The symbol ^*^ is indicating a p < 0.05 with the comparison of group 1 vs. group 2; the symbol

** is indicating the p < 0.05 with the comparison of group 1 vs. group 3; the symbol

*** is indicating a p < 0.05 with the comparison of group 2 vs. group 3.

*The symbol / is indicating a not statistically significant value. The p < 0.05 is indicating a statistically significant p-value*.

### Clinical characteristics of obese patients with pre-diabetic condition treated by a hypocaloric diet added to metformin (group 1) at 12 months of follow-up vs. baseline

At 12 months follow-up as compared to admission values, group 1 of patients experienced a statistically significant reduction of cholesterol (4.04 ± 0.81 vs. 4.33 ± 0.86 mmol/L, *p* <0.05), triglycerides (1.66 ± 0.96 vs. 1.89 ± 0.44 mmol/L, *p* <0.05), and glucose blood values (5.48 ± 0.12 vs. 6.64 ± 0.14 mmol/L, *p* <0.05), associated with higher levels of insulin (22.7 ± 1.7 vs. 19.7 ± 1.9 μU/ml, *p* <0.05), and to an amelioration in HOMA-IR (4.2 ± 0.35 vs. 5.1 ± 0.72, *p* <0.05), and a reduction of WHR (0.79 ± 0.003 vs. 0.91 ± 0.006, *p* <0.05) Table 2. Conversely, patients of group 1 experienced at 12 months of follow-up vs. baseline condition a statistically significant reduction of CRP (0.49 ± 0.02 vs. 1.04 ± 0.48 mmol/L, *p* <0.05), IL6 (3.33 ± 0.34 vs. 4.22 ± 0.45 pg/ml, *p* <0.05), TNFα (5.15 ± 0.44 vs. 6.95 ± 0.59 pg/ml, *p* <0.05), and of Nitrotyrosine (2,151 ± 0,351 vs. 5,214 ± 0,702 nmol/l, *p* <0.05) Table 2. In addition, patients of group 1 experienced at 12 months of follow-up vs. baseline condition a statistically significant reduction of IMT (0.86 ± 0.15 vs. 1.01 ± 0.15 mm, *p* <0.05), septum thickness (10.5 ± 2 vs. 14 ± 2.5 mm, *p* <0.05), posterior wall thickness (8 ± 1 vs. 11 ± 1.5 mm, *p* <0.05), left ventricle mass (133.2 ± 37.6 vs.192.5 ± 49.5 g, *p* <0.05), and of MPI (0.38 ± 0.02 vs. 0.58 ± 0.03, *p* <0.05) Table 2.

**Table 2:** Obese patients with pre-diabetic condition treated by a hypocaloric diet added to metformin (group 1) at baseline vs. 12 months of follow-up.

**Study variables**	**Group 1 (n 20) at baseline**	**Group 1 (n 20) at 12 months of follow-up**	***P*-value**
**CLINICAL VARIABLES**			
BMI (Kg/m^2^)	33.1 ± 2.7	31.9 ± 0.7	/
Systolicarterial pressure (mmHg)	133 ± 11	127 ± 9	/
Diastolicarterial pressure (mmHg)	82 ± 2.3	78 ± 2.5	/
Heart rate (beats for minute)	72 ± 9	67 ± 8	/
WHR	0.91 ± 0.006	0.79 ± 0.003	<0.05^‡^
HOMA-IR	5.1 ± 0.72	4.2 ± 0.35	<0.05^‡^
Insulin(μU/ml)	19.7 ± 1.9	22.7 ± 1.7	<0.05^‡^
Glucose (mmol/L)	6.64 ± 0.14	5.48 ± 0.12	<0.05^‡^
Cholesterol(mmol/L)	4.33 ± 0.86	4.04 ± 0.81	<0.05^‡^
HDL(mmol/L)	1.73 ± 0.44	1.81 ± 0.41	/
LDL (mmol/L)	3.31 ± 0.61	3.11 ± 0.56	/
Triglycerides(mmol/L)	1.89 ± 0.44	1.66 ± 0.96	<0.05^‡^
Creatinine (mmol/L)	98.6 ± 4.4	103.5 ± 6.2	/
**BIOHUMORAL MARKERS**			
CRP (mmol/L)	1.04 ± 0.48	0.49 ± 0.02	<0.05^‡^
IL6(pg/ml)	4.22 ± 0.45	3.33 ± 0.34	<0.05^‡^
TNFα (pg/ml)	6.95 ± 0.59	5.15 ± 0.44	<0.05^‡^
Nitrotyrosine (nmol/l)	5, 214 ± 0, 702	2, 151 ± 0, 351	<0.05^‡^
**ECHOCARDIOGRAPHICPARAMETERS**			
Intima-media tickness (mm)	1.01 ± 0.15	0.86 ± 0.15	<0.05^‡^
LVTDd (mm)	56 ± 3.8	55 ± 3.1	/
LVTSd (mm)	34 ± 4.4	31 ± 5.4	/
LVEF (%)	54 ± 6	56 ± 6	/
LAD (mm)	45 ± 6	44 ± 4	/
Septum (mm)	14 ± 2.5	10.5 ± 2	<0.05^‡^
Posteriorwall (mm)	11 ± 1.5	8 ± 1	<0.05^‡^
MPI	0.58 ± 0.03	0.38 ± 0.02	< 0.05^‡^
LV mass (g)	192.5 ± 49.5	133.2 ± 37.6	<0.05^‡^
LV mass/BSA (g/m^2^)	84.06 ± 21.62	58.16 ± 16.42	<0.05^‡^
LV mass/h (m^2^)	69 ± 17.75	47.74 ± 13.47	<0.05^‡^

Clinical characteristics of obese pre-diabetics (group 1) at baseline vs. 12 months of follow-up. In this table are reported the study variables of groups 1, 20 obese patients with pre-diabetic condition treated by a hypocaloric diet added to metformin. In the study the variables (first column to the left) are reported clinical variables, biohumoral markers, and echocardiographic parameters for each group of patients. We reported fasting glucose and lipid values. CRP, C reactive protein; g, grams; m, meters; h, height; HOMA_IR, homeostasis model for the assessment of insulin resistance; IL6, interleukine 6; LAD, left atrium diameter; LV, left ventricle; LVEF, left ventricle ejection fraction; LVTDd, left ventricle telediastolic diameter; LVTDs, left ventricle telesystolic diameter; MPI, myocardium performance index; TNFα, tumor necrosis factor alpha; WHR, waist hip ratio. The symbol

‡ *is indicating a p <0.05 with the comparison of group 1 at baseline vs. group 1 at 12 months follow-up*.

### Clinical characteristics of obese patients with pre-diabetic condition treated by a hypocaloric diet added to placebo (group 2) at 12 months of follow-up vs. baseline

The patients of group 2 at 12 months of follow-up when compared to admission values experienced a significant reduction of cholesterol (4.15 ± 0.94 vs. 4.51 ± 0.88 mmol/L, *p* <0.05), and of triglyceride blood values (1.71 ± 1.18 vs. 1.83 ± 0.54 mmol/L, *p* <0.05) Table 3.

**Table 3:** Obese pre-diabetic patient treated by a hypocaloric diet added to the placebo (group 2) at baseline vs. 12 months of follow-up.

**Study variables**	**Group 2 (n 20) at baseline**	**Group 2 (n 20) at 12 months of follow-up**	***P*-value**
**CLINICAL VARIABLES**			
BMI (Kg/m^2^)	33.5 ± 2.6	32.3 ± 0.9	/
Systolic arterial pressure (mmHg)	129 ± 12	125 ± 11	/
Diastolicarterial pressure (mmHg)	72 ± 10	77 ± 2.2	/
Heart rate (beats for minute)	84 ± 2.1	71 ± 5	/
WHR	0.91 ± 0.005	0.88 ± 0.005	/
HOMA-IR	4.9 ± 0.68	5.0 ± 0.68	/
Insulin(μU/ml)	20.1 ± 1.8	20.1 ± 1.5	/
Glucose (mmol/L)	6.73 ± 0.24	6.68 ± 0.2	/
Cholesterol(mmol/L)	4.51 ± 0.88	4.15 ± 0.94	<0.05^‡^
HDL(mmol/L)	1.83 ± 0.39	1.84 ± 0.4	/
LDL(mmol/L)	3.33 ± 0.57	3.19 ± 0.57	/
Triglycerides(mmol/L)	1.83 ± 0.54	1.71 ± 1.18	<0.05^‡^
Creatinine (mmol/L)	101.2 ± 3.5	99.4 ± 3.5	/
**BIOHUMORAL MARKERS**			
CRP (mmol/L)	1.03 ± 0.43	0.57 ± 0.03	/
IL6(pg/ml)	4.10 ± 0.39	4.05 ± 0.27	/
TNFα (pg/ml)	6.75 ± 0.53	6.55 ± 0.53	/
Nitrotyrosine (nmol/l)	5, 092 ± 0, 651	4, 511 ± 0, 251	/
**ECHOCARDIOGRAPHICPARAMETERS**			
Intima-media tickness (mm)	1.03 ± 0.18	1.01 ± 0.16	/
LVTDd (mm)	55 ± 4.1	55 ± 2.1	/
LVTSd (mm)	32 ± 4.8	32 ± 3.9	/
LVEF (%)	54 ± 7	54 ± 7	/
LAD (mm)	43 ± 6	43 ± 2	/
Septum (mm)	14 ± 2.2	13.5 ± 2	/
Posteriorwall (mm)	11 ± 1	10.5 ± 1	/
MPI	0.57 ± 0.03	0.49 ± 0.02	/
LV mass (g)	191.7 ± 49.7	178.3 ± 41.1	/
LV mass/BSA (g/m^2^)	82.62 ± 21.42	76.85 ± 17.72	/
LV mass/h (m^2^)	67.03 ± 17.38	62.34 ± 14.37	/

Clinical characteristics of obese patients with pre-diabetic condition (group 2) at baseline vs. 12 months of follow-up. In this table are reported the study variables of groups 2, 20 obese patients with pre-diabetic condition treated by a hypocaloric diet added to the placebo. In the study variables (first column to the left) are reported clinical variables, biohumoral markers, and echocardiographic parameters for each group of patients. We reported fasting glucose and lipid values. CRP, C reactive protein; g, grams; m, meters; h, height; HOMA_IR, homeostasis model for the assessment of insulin resistance; IL6, interleukine 6; LAD, left atrium diameter; LV, left ventricle; LVEF, left ventricle ejection fraction; LVTDd, left ventricle telediastolic diameter; LVTDs, left ventricle telesystolic diameter; MPI, myocardium performance index; TNFα, tumor necrosis factor alpha; WHR, waist hip ratio. The symbol

‡ *is indicating the p <0.05 by the comparison of group 2 at baseline vs. group 2 at 12 months follow-up*.

### Clinical characteristics of obese patients with normo-glycemic condition treated by a hypocaloric diet (group 3) at 12 months of follow-up vs. baseline

Obese patients with a normo-glycemic condition (group 3) experienced, in the 12th month of their follow-up, a significant reduction of cholesterol blood levels in their baseline condition a significant reduction of cholesterol blood levels (4.14 ± 0.81 vs. 4.66 ± 1.02 mmol/L, *p* <0.05), and of inflammatory markers such as CRP (0.48 ± 0.01 vs. 0.85 ± 0.38 mmol/L, *p* <0.05), IL6(3.13 ± 0.43 vs. 3.53 ± 0.43 pg/ml, *p* <0.05), TNFα (4.76 ± 0.79 vs.5.51 ± 1.09 pg/ml, *p* <0.05), and of nitrotyrosine (0,917 ± 0,251 vs. 1,211 ± 0,205 nmol/l, *p* <0.05) Table 4. In addition, obese patients with normo-glycemic condition (group 3) experienced at 12 months of follow-up vs. baseline condition a significant reduction of septum thickness (10 ± 2 vs. 13.5 ± 2.6 mm, *p* <0.05), posterior wall thickness (8.5 ± 1 vs. 11 ± 1 mm, *p* <0.05), left ventricle mass (129.2 ± 33.1 vs. 203.7 ± 48.4 grams, *p* <0.05), and of MPI (0.37 ± 0.02 vs. 0.57 ± 0.03, *p* <0.05) Table 4.

**Table 4:** Obese patients with normo-glycemic condition treated by a hypocaloric diet (group 3).

**Study variables**	**Group 3 (n 18) at baseline**	**Group 3 (n 18) at 12 months of follow-up**	***P*-value**
**CLINICAL VARIABLES**			
BMI (Kg/m^2^)	33.7 ± 2.4	31.7 ± 0.6	/
Systolicarterial pressure (mmHg)	126 ± 10.3	124 ± 8.8	/
Diastolicarterial pressure (mmHg)	85 ± 2.1	75 ± 2.3	/
Heart rate (beats for minute)	69 ± 8	66 ± 7	/
WHR	0.91 ± 0.001	0.81 ± 0.001	/
HOMA-IR	4.1 ± 0.28	4.1 ± 0.28	/
Insulin(μU/ml)	22.6 ± 1.9	23.1 ± 1.6	/
Glucose (mmol/L)	5.34 ± 0.57	5.32 ± 0.59	/
Cholesterol(mmol/L)	4.66 ± 1.02	4.14 ± 0.81	<0.05°
HDL(mmol/L)	1.78 ± 0.41	1.78 ± 0.41	/
LDL (mmol/L)	3.17 ± 0.59	3.11 ± 0.57	/
Triglycerides(mmol/L)	1.61 ± 0.31	1.62 ± 0.67	/
Creatinine (mmol/L)	78.3 ± 2.6	74.8 ± 1.8	/
**BIOHUMORAL MARKERS**			
CRP (mmol/L)	0.85 ± 0.38	0.48 ± 0.01	<0.05°
IL6 (pg/ml)	3.53 ± 0.43	3.13 ± 0.43	<0.05°
TNFα (pg/ml)	5.51 ± 1.09	4.76 ± 0.79	<0.05°
Nitrotyrosine (nmol/l)	*1, 211*±*0, 205*	*0, 917*±*0, 251*	<0.05°
**ECHOCARDIOGRAPHICPARAMETERS**			
Intima-media tickness (mm)	0.85 ± 0.14	0.83 ± 0.15	/
LVTDd (mm)	54 ± 4.6	53 ± 4.1	/
LVTSd (mm)	31 ± 6.7	29 ± 6.7	/
LVEF (%)	53 ± 6	55 ± 5	/
LAD (mm)	42 ± 2	41 ± 2	/
Septum (mm)	13.5 ± 2.6	10 ± 2	<0.05°
Posteriorwall (mm)	11 ± 1	8.5 ± 1	<0.05°
MPI	0.57 ± 0.03	0.37 ± 0.02	<0.05°
LV mass (g)	203.7 ± 48.4	129.2 ± 33.1	<0.05°
LV mass/BSA (g/m^2^)	90.13 ± 21.42	57.17 ± 14.64	<0.05°
LV mass/h (m^2^)	72.23 ± 17.16	45.81 ± 11.73	<0.05°

Clinical characteristics of obese patients with normo-glycemic condition (group 3) at baseline vs. 12 months of follow-up. In this table are reported the study variables of group 3, 18 obese patients with normo glycemic condition treated by a hypocaloric diet. In the study the variables (first column to the left) reported are clinical variables, biohumoral markers, and echocardiographic parameters for each group of patients. We reported fasting glucose and lipid values. CRP, C reactive protein; g, grams; m, meters; h, height; HOMA_IR, homeostasis model for the assessment of insulin resistance; IL6, interleukine 6; LAD, left atrium diameter; LV, left ventricle; LVEF, left ventricle ejection fraction; LVTDd, left ventricle telediastolic diameter; LVTDs, left ventricle telesystolic diameter; MPI, myocardium performance index; TNFα, tumor necrosis factor alpha; WHR, waist hip ratio. The symbol symbol

° *is indicating the p <0.05 with the comparison of group 3 at baseline vs. group 3 at 12 months follow-up*.

### Clinical characteristics and comparison between the three groups of patients at 12 months of follow-up

In the 12th month of the follow-up, obese patients with pre-diabetics condition treated by metformin (group 1) vs. obese patients with pre-diabetic condition treated by placebo (group 2) experienced a significant reduction of heart rate (67 ± 8 vs. 71 ± 5, *p* <0.05), of WHR (0.79 ± 0.003 vs. 0.88 ± 0.005, *p* <0.05), with a reduction of glucose values (5.48 ± 0.12 vs. 6.68 ± 0.18 mmol/L, *p* <0.05), and an amelioration of insulin resistance (HOMA-IR 4.2 ± 0.35 vs. 5.0 ± 0.68, *p* <0.05; Insulin 22.7 ± 1.7 vs. 20.1 ± 1.5 μU/ml) Table 5. About inflammatory markers, at 12 months of follow-up, obese patients with pre-diabetic condition treated by metformin (group 1) vs. obese patients with pre-diabetic condition treated by placebo (group 2) experienced a significant reduction of CRP (0.49 ± 0.02 vs. 0.57 ± 0.03 mmol/L, *p* <0.05), IL6(3.33 ± 0.34 vs. 4.05 ± 0.27 pg/ml, *p* <0.05), TNFα (5.15 ± 0.44 vs. 6.55 ± 0.53 pg/ml, *p* <0.05), and of nitrotyrosine (2,151 ± 0,351 vs. 4,511 ± 0,251 nmol/L, *p* <0.05) Table 5. Regarding echocardiographic parameters, at 12 months of follow-up, obese patients with pre-diabetic condition treated by metformin (group 1) vs. obese patients with pre-diabetic condition treated by placebo (group 2) experienced a significant reduction of intima-media thickness (0.86 ± 0.15 vs. 1.01 ± 0.16 mm, *p* <0.05), septum thickness (10.5 ± 2 vs. 13.5 ± 2 mm, *p* <0.05), posterior wall thickness (8 ± 1 vs. 10.5 mm, *p* <0.05), left ventricle mass (133.2 ± 37.6 vs. 178.3 ± 41.1 grams, *p* <0.05), and of MPI (0.38 ± 0.02 vs. 0.49 ± 0.02, *p* <0.05) Table 5. At 12 months of follow-up, obese patients with pre-diabetic condition treated by metformin (group 1) vs. obese patients with normo-glycemic condition(group 3) had higher serum values of creatinine (103.5 ± 6.2 vs. 74.8 ± 1.8 mmol/L, *p* <0.05), and of nitrotyrosine (2,151 ± 0,351 vs. 0,917 ± 0,251 nmol/L, *p* <0.05);no other significantly different parameters were found between the two groups Table 5. At 12 months of follow-up, obese patients with pre-diabetic condition treated by placebo (group 2) vs. obese patients with normo-glycemic condition(group 3) had higher values of heart rate (71 ± 5 vs. 66 ± 7, *p* <0.05), of WHR (0.88 ± 0.005 vs. 0.81 ± 0.001 vs., *p* <0.05), of glucose values (6.68 ± 0.18 vs.5.32 ± 0.59 mmol/L, *p* <0.05), with insulin resistance (HOMA-IR 5.0 ± 0.68 vs. 4.1 ± 0.28, *p* <0.05; Insulin 20.1 ± 1.5 vs. 23.1 ± 1.6 μU/ml), and higher serum values of creatinine (99.4 ± 3.5 vs. 74.8 ± 1.8 mmol/L, p <0.05) Table 5. At 12 months of follow-up, obese patients with pre-diabetic condition treated by placebo (group 2) vs. obese patients with normo-glycemic condition (group 3) had higher values of CRP (0.57 ± 0.03 vs. 0.48 ± 0.01 vs. mmol/L, *p* <0.05), IL6(4.05 ± 0.27 vs. 3.13 ± 0.43 pg/ml, *p* <0.05), TNFα (6.55 ± 0.53 vs. 4.76 ± 0.79 pg/ml, *p* <0.05), and nitrotyrosine (4,511 ± 0,251 vs. 0,917 ± 0,251 nmol/L, *p* <0.05) Table 5. Regarding echocardiographic parameters, at 12 months of follow-up, obese patients with pre-diabetic condition treated by placebo (group 2) vs. obese patients with normo-glycemic condition (group 3) had higher values of intima-media thickness (1.01 ± 0.16 vs. 0.83 ± 0.15 mm, *p* <0.05), septum thickness (13.5 ± 2 vs. 10 ± 2 mm, *p* <0.05), posterior wall thickness (10.5 vs. 8.5 ± 1 mm, *p* <0.05), left ventricle mass (178.3 ± 41.1 vs. 129.2 ± 33.1 grams, p <0.05), and MPI (0.49 ± 0.02 vs. 0.37 ± 0.02 *p* <0.05) Table 5.

**Table 5:** Clinical characteristics of the study population at 12 months of follow-up.

**Study variables**	**Group 1 (n 20)**	**Group 2 (n 20)**	**Group 3 (n 18)**	***P*-value (group 1 vs. group 2)**	***P*-value (group 1 vs. group 3)**	***P*-value (group 2 vs. group 3)**
**CLINICAL VARIABLES**						
Systolicarterial pressure (mmHg)	127 ± 9	125 ± 11	124 ± 8.8	/	/	/
Diastolicarterial pressure (mmHg)	78 ± 2.5	77 ± 2.2	75 ± 2.3	/	/	/
Heart rate (beats for minute)	67 ± 8	71 ± 5	66 ± 7	<0.05^*^	/	<0.05^***^
BMI (Kg/m^2^)	31.9 ± 0.7	32.3 ± 0.9	31.7 ± 0.6	/	/	/
WHR	0.79 ± 0.003	0.88 ± 0.005	0.81 ± 0.001	<0.05^*^	/	<0.05^***^
HOMA-IR	4.2 ± 0.35	5.0 ± 0.68	4.1 ± 0.28	<0.05^*^	/	< 0.05^***^
Insulin(μU/ml)	22.7 ± 1.7	20.1 ± 1.5	23.1 ± 1.6	<0.05^*^	/	<0.05^***^
Glucose (mmol/L)	5.48 ± 0.12	6.68 ± 0.18	5.32 ± 0.59	<0.05^*^	/	<0.05^***^
Cholesterol (mmol/L)	4.04 ± 0.81	4.15 ± 0.94	4.14 ± 0.81	/	/	/
HDL (mmol/L)	1.81 ± 0.41	1.84 ± 0.4	1.78 ± 0.41	/	/	/
LDL (mmol/L)	3.11 ± 0.56	3.19 ± 0.57	3.11 ± 0.57	/	/	/
Triglycerides(mmol/L)	1.66 ± 0.96	1.71 ± 1.18	1.62 ± 0.67	/	/	/
Creatinine (mmol/L)	103.5 ± 6.2	99.4 ± 3.5	74.8 ± 1.8	/	<0.05^**^	<0.05^***^
**BIOHUMORAL MARKERS**						
CRP (mmol/L)	0.49 ± 0.02	0.57 ± 0.03	0.48 ± 0.01	<0.05^*^	/	<0.05^***^
IL6 (pg/ml)	3.33 ± 0.34	4.05 ± 0.27	3.13 ± 0.43	<0.05^*^	/	<0.05^***^
TNFα (pg/ml)	5.15 ± 0.44	6.55 ± 0.53	4.76 ± 0.79	<0.05^*^	/	<0.05^***^
Nitrotyrosine (nmol/l)	2, 151 ± 0, 351	4, 511 ±0, 251	0, 917 ± 0, 251	<0.05^*^	<0.05^**^	<0.05^***^
**ECHOCARDIOGRAPHICPARAMETERS**						
Intima-media tickness	0.86 ± 0.15	1.01 ± 0.16	0.83 ± 0.15	<0.05^*^	/	<0.05^***^
LVTDd (mm)	55 ± 3.1	55 ± 2.1	53 ± 4.1	/	/	/
LVTSd (mm)	31 ± 5.4	32 ± 3.9	29 ± 6.7	/	/	/
LVEF (%)	56 ± 6	54 ± 7	55 ± 5	/	/	/
LAD (mm)	44 ± 4	43 ± 2	41 ± 2	/	/	/
Septum (mm)	10.5 ± 2	13.5 ± 2	10 ± 2	<0.05^*^	/	<0.05^***^
Posteriorwall (mm)	8 ± 1	10.5 ± 1	8.5 ± 1	<0.05^*^	/	<0.05^***^
MPI	0.38 ± 0.02	0.49 ± 0.02	0.37 ± 0.02	<0.05^*^	/	<0.05^***^
LV mass	133.2 ± 37.6	178.3 ± 41.1	129.2 ± 33.1	<0.05^*^	/	<0.05^***^
LV mass/BSA (g/m^2^)	58.16 ± 16.42	76.85 ± 17.72	57.17 ± 14.64	<0.05^*^	/	<0.05^***^
LV mass/h (m^2^)	47.74 ± 13.47	62.34 ± 14.37	45.81 ± 11.73	<0.05^*^	/	<0.05^***^

Clinical characteristics of the study population as obese patients with pre-diabetic condition (groups 1 and 2), and obese patients with normo-glycemic condition (group 3) at 12 months of follow-up. In this table are reported the study variables of the study population of 58 patients divided into three groups at 12 months of follow-up: group 1, 20 pre-diabetics obese patients treated by hypocaloric diet added to metformin; group 2, 20 patients with pre-diabetic condition treated by a hypocaloric diet added to placebo; group 3, 18 patients with normo-glycemic condition treated by a hypocaloric diet. In the study variables (first column to the left) are reported clinical variables, biohumoral markers, and echocardiographic parameters for each group of patients. . We reported fasting glucose and lipid values. CRP, C reactive protein; g, grams; m, meters; h height; HOMA_IR, homeostasis model for the assessment of insulin resistance; IL6, interleukine 6; LAD, left atrium diameter; LV, left ventricle; LVEF, left ventricle ejection fraction; LVTDd, left ventricle telediastolic diameter; LVTDs, left ventricle telesystolic diameter; MPI, myocardium performance index; TNFα tumor necrosis factor alpha; WHR, waist hip ratio. The symbol

* is indicating a p <0.05 with the comparison of group 1 vs. group 2; the symbol

** is indicating the p <0.05 with the comparison of group 1 vs. group 3; the symbol

*** *is indicating the p <0.05 with the comparison of group 2 vs. group 3. The symbol/is indicating a not statistically significant value. The p <0.05 is indicating a statistically significant p-value*.

### Regression analysis results: relation between the study variables and the clinical study outcomes

As shown in Table 6, IL6 expression was related to higher values of LV mass (*R*-value 0.272, *p*-value 0.039), and to higher IMT (*R*-value 0.272, *p*-value 0.039), such as those observed for C reactive protein (CRP) (*R*-value 0.308, *p*-value 0.021), for glucose blood values (*R*-value 0.449, *p*-value 0.001), and for HOMA-IR (*R*-value 0.366, *p*-value 0.005). On the contrary, an inverse correlation was found between fat tissue SIRT1 values and MPI (*R*-value−0.236, *p*-value 0.002).

**Table 6:** In the table linear regression analysis for study variables and study outcomes.

**Variables**	**Δ LV MASS**		**Δ LVEF**		**Δ MPI**		**Δ IMT**	
	***R*-value**	***P*-value**	***R*-value**	***P*-value**	***R*-value**	***P*-value**	***R*-value**	***P*-value**
CRP	0.251	0.058	0.018	0.892	0.211	0.112	0.308	0.021^*^
IL6	0.272	0.039^*^	0.147	0.270	0.226	0.088	0.272	0.039^*^
TNFα	0.147	0.271	0.165	0.216	0.087	0.517	0.147	0.271
SIRT1	0.041	0.763	0.166	0.215	−0.236	0.002^*^	0.041	0.763
Cholesterol	0.162	0.223	0.160	0.230	0.229	0.083	0.162	0.223
Creatinine	0.180	0.176	0.232	0.080	0.162	0.224	0.180	0.176
Insulin	0.159	0.233	0.036	0.791	0.060	0.652	0.023	0.865
HOMA-IR	0.114	0.393	0.021	0.877	0.092	0.493	0.366	0.005^*^
Systolicarterial pressure	0.009	0.949	0.212	0.110	0.201	0.133	0.009	0.949
Glucose	0.162	0.225	0.081	0.545	0.007	0.951	0.449	0.001^*^

In the table is seen linear regression analysis for study variables and study outcomes. For each study outcome the R-value and the p value are reported. The study outcomes are expressed as delta (Δ)-values, that represent changes between follow-up vs. baseline values. The symbol

* *is indicating a p <0.05. The p <0.05 is indicating a statistically significant p-value. CRP, C reactive protein; HOMA_IR, homeostasis model for the assessment of insulin resistance; IL6, interleukine 6; MPI, myocardium performance index; SIRT1, sirtuin 1; TNFα tumor necrosis factor alpha*.

## Discussion

The novelty of this study is that, in patients with pre-diabetic condition vs. patients with normo-glycemic condition, the baseline hyperglycemic condition and the insulin resistance are linked to higher expression of serum inflammatory cytokines and nitrotyrosine, and to a lower expression of SIRT1 in the subcutaneous abdominal fat Figures 1–6, Tables 1–5. Numerous findings may explain the link between inflammation, oxidative stress, and prediabetes. For example, the adipose tissue Fas over expression in obese patients with prediabetes vs. patients with normo-glycemic condition (group 1 and group 2 vs. group 3) is a marker of insulin resistance, apoptosis, and inflammation (Blüher et al., 2014) Table 1, Figure 2. In adipocytes of mice model, the deletion of the death receptor Fas is associated with improved insulin sensitivity and reduced adipose tissue inflammation (Blüher et al., 2014). Independently of body weight, increased Fas expression may contribute to impaired insulin sensitivity and adipose tissue dysfunction in obesity (Blüher et al., 2014). Parallel to the adipose tissue Fas over expression, in the present study patients with pre-diabetic condition in both groups presented at baseline higher values of adipose tissue and serum nitrotyrosine as compared to patients with normo-glycemic condition Table 1, Figure 5. Nitrotyrosine is produced by the oxidation of tyrosine, and is an index of oxidative stress in overweight patients (Sardu et al., 2014), as well as in patients with diabetes (Giugliano et al., 1996). Indeed, hyperglycemia is directly involved in the subsequent nitrotyrosine formation in patients with diabetes (Giugliano et al., 1996). In addition, patients with prediabetic condition vs. patients with normo-glycemic condition expressed at baseline a statistically significantly lower value of SIRT1, that is down regulated by altered glucose homeostasis, and linked to an altered myocardial performance (Huang et al., 2016; Rappou et al., 2016) Table 1, Figure 1. These molecular inflammatory/oxidative pathways were linked to different echographic alterations. In fact, patients with pre-diabetic condition vs. obese patients with normo-glycemic condition had higher intima-media thickness values at baseline Table 1. Intima-media thickness is an important atherosclerotic risk marker, and it is due to an adaptive response to changes in flow, wall tension, or lumen diameter (Stein et al., 2008). On other hand, the higher intima-media thickness may be also be the result of non-atherosclerotic processes such as smooth muscle cell hyperplasia and fibro-cellular hypertrophy, leading to medial hypertrophy and compensatory arterial remodeling (Stein et al., 2008). However, obese patients with prediabetic condition may have anatomic and physiologic changes, that are consistent with early arterial disease (Houtkooper et al., 2012). At baseline in patients with pre-diabetic condition vs. normo-glycemic condition these molecular inflammatory/oxidative alterations were linked to higher values of septum thickness, posterior wall, left ventricle mass, and MPI Table 1. These molecular and echographic alterations are due to multiple cardiovascular risk factors, and additionally to prediabetes (Houtkooper et al., 2012). In this setting, at 12 months of follow-up the metformin therapy vs. placebo determined a significant reduction of glucose blood levels and of the HOMA-IR, with an increase of insulin blood levels Table 5. These effects were associated with a significant reduction of inflammatory and oxidative stress markers, such as CRP, IL6, TNFα, and nitrotyrosine Table 2. On other hand, obese patients with prediabetic condition treated with placebo in addition to a hypocaloric diet experienced only a reduction of cholesterol, and of triglyceride blood values, with no effect on inflammatory and oxidative stress markers Table 3. On the contrary, the patients with normo-glycemic condition (group 3) treated by a hypocaloric diet, experienced at 12 months of follow-up vs. baseline condition a significant reduction of cholesterol blood levels, and of inflammatory/oxidative stress markers Table 4. Therefore, in obese patients with normo-glycemic condition the hypocaloric diet was enough to reduce inflammation and oxidative stress, and this was associated with a significant reduction of septum thickness, posterior wall, and left ventricle mass Table 4. All these effects were similar to those induced by metformin therapy in obese patients with prediabetic condition Table 2. Consequently, in the study population higher values of CRP, serum glucose, and HOMA-IR were linked to higher values of IMT Table 6. Furthermore, the significant reduction of IL6 induced by metformin, may be related to higher values of LV mass and IMT values Table 6. Intriguingly, an inverse correlation may exist between adipose tissue SIRT1 expression and the MPI values (*R*-value −0.236, *p*-value 0.002) Table 6. However, we may say that, higher adipose tissue SIRT1 values may be linked to better cardiac performance (MPI reduction). All these results may confirm a link between inflammation, oxidative stress, and cardiac performance in obese pre-diabetics, and its possible modulation induced by metformin. Entering into the merits of the discussion, IL6 is a complex protein and an inflammatory marker related to oxidative stress, and induced by hyperglycemia (Shah et al., 2014). The up-regulation of IL6 is linked to the development of LV hypertrophy in patients with diabetics (Gregersen et al., 2012), and in obese patients (Palmieri et al., 2003). In our study the LV mass was reduced by metformin therapy in addition to a hypocaloric diet, as compared to placebo, and this was associated with a significant down-regulation of IL6 in obese patients with pre-diabetic condition at the 12th month of treatment Table 2. IL6 is also correlated to IMT, because the IL6 over expression induces cardiac cellular growth and cardiac hypertrophy (Palmieri et al., 2003). Consequently, we may speculate that, hyperglycemic stress may induce complex alterations of molecular pathways and cellular receptors, with an alteration in mitochondrial respiration, cellular functions, and cardiac performance (Abel et al., 2008). Secondly, in obese patients with pre-diabetic condition the metformin therapy may also reduce the oxidative stress (Abel et al., 2008). Indeed, in our study we reported a significant reduction of serum nitrotyrosine values in obese patients with pre-diabetic condition treated by metformin vs. placebo Table 2, 5. However, the hyperglycemia and insulin resistance phenotype may induce in obese patients with pre-diabetic condition an over activation of inflammation, and of oxidative stress (Wellen and Hotamisligil, 2005; Ford et al., 2010; Zhang et al., 2015), and this may consequently cause higher values of IMT and cardiac dysfunction Table 3. Numerous findings may explain this correlation between inflammation, oxidative stress, and cardiac function in obese patients with pre-diabetic condition. To date, firstly the inflammatory axis is a well-known cause of advanced oxidation and heart dysfunction in obese patients (Wang and Nakayama, 2010), and in patients with pre-diabetic condition (Ford et al., 2010). Secondly, SIRT1 may represent a key mediator directly involved in cardiac remodeling during pathologically adaptive conditions (Kehat and Molkentin, 2010; Matsushima and Sadoshima, 2015). In this study, we reported a cardiac remodeling by an increase in septum wall thickness, posterior wall thickness, and myocardial mass, and then conditioning of the systolic and diastolic cardiac function, as assessed by the MPI (Marfella et al., 2009). MPI is a Doppler index of combined systolic and diastolic function (de Simone et al., 1992; Marfella et al., 2009), derived from aortic and mitral flows Doppler velocities, and related to worse prognosis in stable and unstable cardiac diseases (Dujardin et al., 1998; Poulsen et al., 2000). Indeed, in obese patients MPI changes are linked to the over activation of subcutaneous abdominal fat inflammation, leading to the reduction of the cardiac contractile function (Marfella et al., 2009). In our study we reported these results in pre-diabetics obese patients, and SIRT1 may represent a key protein linking these inflammatory pathways to cardiac remodeling processes. In fact, SIRT1 may work as a cross-talking effector between adipose tissue and systemic inflammation/oxidative stress, and heart remodeling processes (Matsushima and Sadoshima, 2015). In an animal model of diabetes, the SIRT1 is expressed in pancreatic β-cells to enhance insulin secretion in response to glucose (Moynihan et al., 2005). In detail, SIRT1 is involved in insulin secretion, increased ATP production, and enhanced insulin secretion during glucose stimulation (Moynihan et al., 2005). Consequently, SIRT1 activators inhibit vacuolization, degeneration, and inflammation in the heart of patients with insulin resistance (Sundaresan et al., 2011). However, SIRT1 activators play an anti-remodeling effect during adaptive cardiac hypertrophy (Sundaresan et al., 2011). In our study, in obese patients with pre-diabetic condition the cardiac hypertrophy and the cardiac dysfunction may be linked to reduced adipose tissue levels of SIRT1. As reported by us, metformin therapy in addition to hypocaloric diet vs. placebo reduced the inflammation and oxidative stress Tables 2, 3, 5. Metformin is a well-known hypoglycemic drug in insulin sensitive and insulin resistant obese patients (Seifarth et al., 2013). Metformin therapy may reduce inflammatory status and atherosclerosis by a direct SIRT1 induction (Xu et al., 2015). This effect may be the result of different and numerous functions that metformin induced. Recent data suggest, that metformin alleviates hepatic steatosis through kinase-independent and SIRT1-mediated effects on the autophagy machinery (Song et al., 2015). To date, the addition of metformin to the hypocaloric diet in the obese patients with pre-diabetic condition may result in a reduced expression of serum inflammatory cytokines and nitrotyrosine, and this may be linked to a positive modulation of SIRT1 (Seifarth et al., 2013; Song et al., 2015; Xu et al., 2015). However, metformin therapy may reduce the inflammatory/oxidative stress with a direct SIRT1 induction in obese patients with prediabetic condition (Moynihan et al., 2005; Sundaresan et al., 2011; Seifarth et al., 2013; Song et al., 2015; Xu et al., 2015). All these effects may reduce intima-media thickness, myocardial wall thickness, and myocardial mass, leading to the improvement of the cardiac function in obese patients with pre-diabetic condition. Conversely, we may say that, diet therapy alone may be not effective in obese patients with prediabetic condition. On the contrary, in the healthy obese patients the hypocaloric diet works just as well, to reduce inflammation/oxidative stress, and to improve cardiac performance. Obesity is a major risk factor for hypertension, peripheral arterial disease, coronary artery disease, and heart failure (Lavie et al., 2009). In fact, obese patients with normo-glycemic condition over express inflammatory and pro-oxidative molecules (Alpert, 2001). This is associated with the reduction of myocardial performance (Romero-Corral et al., 2008). However, in obese patients with normo-glycemic condition the hypocaloric diet therapy may reduce the over expression of inflammatory and oxidative stress markers, and this may consequently result in the amelioration of echographic parameters and cardiac performance (Lavie et al., 2009).

## Conclusion

Abdominal fat tissue in obese patients with pre-diabetic condition is a relevant source of inflammatory and oxidative stress metabolites, such as inflammatory cytokines and nitrotyrosine. In our study, the over expression of these molecules is associated at baseline to the adipose tissue hypo expression of SIRT1. This may consequently lead to local and systemic effects, that may affect the cardiac performance in patients with prediabetic condition. On the other hand, we may speculate that, the down regulation of inflammation and oxidative stress may up regulate adipose tissue SIRT1 expression. In this setting, in obese patients with pre-diabetic condition the metformin therapy may reduce inflammatory cytokines and nitrotyrosine serum values. This anti-inflammatory/anti oxidative effect may be associated with a possible metformin induced SIRT1 modulation. Consequently, the metformin therapy induced anti-inflammatory/anti oxidative effects in addition to a SIRT1 regulation (Song et al., 2015; Xu et al., 2015), may lead to the reduction of ITM, and other echocardiographic parameters, such as septum and posterior wall thickness, LV mass, and MPI. However, we may speculate that, adipose tissue SIRT1 may be involved in anti-remodeling cardiac effects in patients with pre-diabetic condition. Consequently, metformin therapy reducing hyperglycemia and insulin resistance, may revert the systemic inflammation/oxidative stress in obese patients with prediabetic condition with SIRT1 modulation (Seifarth et al., 2013; Song et al., 2015; Xu et al., 2015). All these metformin induced therapeutic effects may lead to an amelioration of cardiac performance. Future studies will assess this metformin induced cardiac effect in obese patients with pre-diabetic condition, and its possible correlation with abdominal fat SIRT1 regulation.

## Study limitations

The limitations of this study are: i) the short duration of follow-up that may affect the long term outcomes; ii) the small sample size of obese patients with pre-diabetic condition that may affect the study results; iii) we did not use animal or cellular models to test the human study results obtained by peripheral blood analysis and by direct analysis of samples by abdominal fat tissue biopsy. Thus, further long-term studies in a larger population of obese patients with pre-diabetic condition will be needed to confirm our findings and to determine if the metformin-induced abdominal fat regulation of SIRT1 and of cytokine blood levels added to abdominoplastic surgery and hypocaloric diet could be translated into a reduced incidence of cardiovascular disease in patients with pre-diabetic condition.

## Ethics statement

This study was carried out in accordance with the recommendations of international guidelines for diagnosis and care of diabetics (and prediabetics) obese patients, committee University of Campania Luigi Vanvitelli. The protocol was approved by the committee University of Campania Luigi Vanvitelli. All subjects gave written informed consent in accordance with the Declaration of Helsinki. This study was carried out in accordance with the recommendations of international guidelines, committee University of Campania Luigi Vanvitelli.

## Author contributions

CS: study design, statistical analysis and wrote the manuscript. GP: study design. ND: figures and tables. FC and GN: surgical interventions. PP: study design. MP, MC, MR, AG, and NP: study revision. RM, MB, and MLB: study revision and editing. PM and AD: data collection. FF and IP: immunoistocemistry, and figures.

### Conflict of interest statement

The authors declare that the research was conducted in the absence of any commercial or financial relationships that could be construed as a potential conflict of interest. The reviewer JX and handling editor declared their shared affiliation at the time of the review.

## References

[B1] Abel E. D. Litwin S. E. Sweeney G. (2008). Cardiac remodeling in obesity. Physiol. Rev. 88, 389–419. 10.1152/physrev.00017.2007 18391168 PMC2915933

[B2] Alpert M. A. (2001). Obesity cardiomyopathy: pathophysiology and evolution of the clinical syndrome. Am. J. Med. Sci. 321, 225–236. 10.1097/00000441-200104000-00003 11307864

[B3] Blüher M. Klöting N. Wueest S. Schoenle E. J. Schön M. R. Dietrich A.. (2014). Fas and FasL expression in human adipose tissue is related to obesity, insulin resistance, and type 2 diabetes. J. Clin. Endocrinol. Metab. 99, E36–E44. 10.1210/jc.2013-2488 24178789

[B4] de Simone G. Daniels S. R. Devereux R. B. Meyer R. A. Roman M. J. de Divitiis O.. (1992). Left ventricular mass and body size in normotensive children and adults: assessment of allometric relations and the impact of overweight. J. Am. Coll Cardiol. 20, 1251–1260. 10.1016/0735-1097(92)90385-Z 1401629

[B5] Dujardin K. S. Tei C. Yeo T. C. Hodge D. O. Rossi A. Seward J. B. (1998). Prognostic value of a Doppler index combining systolic and diastolic performance in idiopathic-dilated cardiomyopathy. Am. J. Cardiol. 82, 1071–1076. 10.1016/S0002-9149(98)00559-1 9817484

[B6] Ford E. S. Zhao G. Li C. (2010). Pre-diabetes and the risk for cardiovascular disease: a systematic review of the evidence. JACC 55, 1310–1317. 10.1016/j.jacc.2009.10.060 20338491

[B7] Giugliano D. Ceriello A. Paolisso G. (1996). Oxidative stress and diabetic vascular complications. Diabetes Care 19, 257–267. 10.2337/diacare.19.3.257 8742574

[B8] Gregersen S. Samocha-Bonet D. Heilbronn L. K. Campbell L. V. (2012). Inflammatory and oxidative stress responses to high-carbohydrate and high-fat meals in healthy humans. J. Nutr. Metab. 2012:238056. 10.1155/2012/238056 22474579 PMC3306970

[B9] Houtkooper R. H. Pirinen E. Auwerx J. (2012). Sirtuins as regulators of metabolism and healths pan. Nat. Rev. Mol. Cell Biol. 13, 225–238. 10.1038/nrm3293 22395773 PMC4872805

[B10] Huang Y. Cai X. Mai W. Li M. Hu Y. (2016). Association between prediabetes and risk of cardiovascular disease and all cause mortality: systematic review and meta-analysis. BMJ. 355:i5953. 10.1136/bmj.i5953 27881363 PMC5121106

[B11] Kehat I. Molkentin J. D. (2010). Molecular pathways underlying cardiac remodeling during pathophysiologic stimulation. Circulation 122:10 10.1161/CIRCULATIONAHA.110.942268 PMC3076218 21173361

[B12] Lang R. M. Badano L. P. Mor-Avi V. Afilalo J. Armstrong A. Ernande L.. (2015). Recommendations for cardiac chamber quantification by echocardiography in adults: an update from the American Society of Echocardiography and the European Association of Cardiovascular Imaging. Eur. Heart J. Cardiovasc. Imaging. 16, 233–271. 10.1093/ehjci/jev014 25712077

[B13] Lavie C. J. Milani R. V. Ventura H. O. (2009). Obesity and Cardiovascular disease: risk factor, paradox, and impact of weight loss. J. Am. Coll. Cardiol. 53, 1925–1932. 10.1016/j.jacc.2008.12.068 19460605

[B14] Marfella R. Grella R. Rizzo M. R. Barbieri M. Grella R. Ferraraccio F.. (2009). Role of subcutaneous abdominal fat on cardiac function and proinflammatory cytokines in premenopausal obese women. Ann Plast. Surg. 63, 490–495. 10.1097/SAP.0b013e3181955cdb 19806043

[B15] Matsushima S. Sadoshima J. (2015). The role of sirtuins in cardiac disease. Am. J. Physiol. Heart Circ. Physiol. 309, H1375–H1389. 10.1152/ajpheart.00053.2015 26232232 PMC4666968

[B16] Moynihan K. A. Grimm A. A. Plueger M. M. Bernal-Mizrachi E. Ford E. Cras-Méneur C.. (2005). Increased dosage of mammalian Sir2 in pancreatic beta cells enhances glucose-stimulated insulin secretion in mice. Cell Metab. 2, 105–117. 10.1016/j.cmet.2005.07.001 16098828

[B17] Palmieri V. Tracy R. P. Roman M. J. Liu J. E. Best L. G. Bella J. N.. (2003). Strong heart study. Relation of left ventricular hypertrophy to inflammation and albuminuria in adults with type 2 diabetes: the strong heart study. Diabetes Care 26, 2764 −2769. 10.2337/diacare.26.10.2764 14514577

[B18] Poulsen S. H. Jensen S. E. Tei C. Seward J. B. Egstrup K. (2000). Value of the Doppler index of myocardial performance in the early phase of acute myocardial infarction. J. Am. Soc. Echocardiogr. 13, 723–730. 10.1067/mje.2000.105174 10936815

[B19] Rappou E. Jukarainen S. Rinnankoski-Tuikka R. Kaye S. Heinonen S. Hakkarainen A.. (2016). Weight loss is associated with increased NAD^(+)^/SIRT1 expression but reduced PARP activity in white adipose tissue. J. Clin. Endocrinol. Metab. 101, 1263–1273. 10.1210/jc.2015-3054 26760174

[B20] Romero-Corral A. Sierra-Johnson J. Lopez-Jimenez F. Thomas R. J. Singh P. Hoffmann M.. (2008). Relationships between leptin and C-reactive protein with cardiovascular disease in the adult general population. Nat. Clin. Pract. Cardiovasc. Med. 5, 418–425. 10.1038/ncpcardio1218 18431365

[B21] Sardu C. Carreras G. Katsanos S. Kamperidis V. Pace M. C. Passavanti M. B.. (2014). Metabolic syndrome is associated with a poor outcome in patients affected by outflow tract premature ventricular contractions treated by catheter ablation. BMC Cardiovasc. Disord. 14:176. 10.1186/1471-2261-14-176 25480761 PMC4364311

[B22] Seifarth C. Schehler B. Schneider H. J. (2013). Effectiveness of metformin on weight loss in non-diabetic individuals with obesity. Exp. Clin. Endocrinol. Diabetes 121, 27–31. 10.1055/s-0032-1327734 23147210

[B23] Shah A. S. Gao Z. Urbina E. M. Kimball T. R. Dolan L. M. (2014). Prediabetes: the effects on arterial thickness and stiffness in obese youth. J. Clin. Endocrinol. Metab. 99, 1037–1043. 10.1210/jc.2013-3519 24423349 PMC3942227

[B24] Song Y. M. Lee Y. H. Kim J. W. Ham D. S. Kang E. S. Cha B. S.. (2015). Metformin alleviates hepatosteatosis by restoring SIRT1-mediated autophagy induction via an AMP-activated protein kinase-independent pathway. Autophagy 11, 46–59. 10.4161/15548627.2014.984271 25484077 PMC4502778

[B25] Sozer S. O. Agullo F. J. Santillan A. A. Wolf C. (2007). Decision making in abdominoplasty. Aesthetic Plast. Surg. 31, 117–127. 10.1007/s00266-006-0148-y 17205254

[B26] Standards of Care (2017). Standards of medical care in diabetes 2017. Diabetes Care 40(Suppl. 1), S1–S2. 10.2337/cd16-0067 27979885

[B27] Stein J. H. Korcarz C. E. Hurst R. T. Lonn E. Kendall C. B. Mohler E. R.. (2008). Use of carotid ultrasound to identify subclinical vascular disease and evaluate cardiovascular disease risk: a consensus statement from the American Society of Echocardiography Carotid Intima-Media Thickness Task Force. J. Am. Soc. Echocardiogr. 21, 93–111. 10.1016/j.echo.2007.11.011 18261694

[B28] Sundaresan N. R. Pillai V. B. Wolfgeher D. Samant S. Vasudevan P. Parekh V.. (2011). The deacetylase SIRT1 promotes membrane localization and activation of Akt and PDK1 during tumorigenesis and cardiac hypertrophy. Sci. Signal. 4:ra46. 10.1126/scisignal.2001465 21775285

[B29] Wang Z. Nakayama T. (2010). Inflammation, a link between obesity and cardiovascular disease. Mediators Inflamm. 2010:535918. 10.1155/2010/535918 20847813 PMC2929614

[B30] Wellen K. E. Hotamisligil G. S. (2005). Inflammation, stress, and diabetes. J. Clin. Invest. 115, 1111–1119. 10.1172/JCI25102 15864338 PMC1087185

[B31] WHO (2000). Obesity: preventing and managing the global epidemic. Report of a WHO consultation. World Health Organ. Tech. Rep. Ser. 894, i–xii. 1–253. 11234459

[B32] Xu W. Deng Y. Y. Yang L. Zhao S. Liu J. Zhao Z.. (2015). Metformin ameliorates the proinflammatory state in patients with carotid artery atherosclerosis through sirtuin 1 induction. Transl. Res. 166, 451–458. 10.1016/j.trsl.2015.06.002 26141671

[B33] Zhang B. Wang J. Xu Y. Zhou X. Liu J. Xu J.. (2015). Correlative association of interleukin-6 with intima media thickness: a meta-analysis. Int. J. Clin. Exp. Med. 8, 4731–4743. 26064414 PMC4443248

